# Impact of induction chemotherapy with concurrent chemoradiotherapy on nasopharyngeal carcinoma: A meta-analysis of randomized controlled trials

**DOI:** 10.3389/fonc.2022.965719

**Published:** 2022-09-13

**Authors:** Ting-Chieh Huang, Chi-Jen Chen, Yi-Fang Ding, Yi-No Kang

**Affiliations:** ^1^ Department of Otorhinolaryngology, Wan Fang Hospital, Taipei Medical University, Taipei, Taiwan; ^2^ Department of Otolaryngology Head and Neck Surgery, Taipei Tzu Chi Hospital, Buddhist Tzu Chi Medical Foundation, New Taipei, Taiwan; ^3^ Evidence-Based Medicine Center, Wan Fang Hospital, Taipei Medical University, Taipei, Taiwan; ^4^ Research Center of Big Data and Meta-analysis Center, Wan Fang Hospital, Taipei Medical University, Taipei, Taiwan; ^5^ Cochrane Taiwan, Taipei Medical University, Taipei, Taiwan; ^6^ Institute of Health Policy & Management, College of Public Health, National Taiwan University, Taipei, Taiwan

**Keywords:** neoadjuvant chemotherapy, concurrent chemoradiotherapy, nasopharynx cancer, induction chemotherapy, nasopharyngeal cancer

## Abstract

**Purpose:**

Induction chemotherapy followed by concurrent chemoradiotherapy (IC-CCRT) may be beneficial for nasopharyngeal carcinoma. However, the evidence on medium- and long-term effects of IC-CCRT is limited, and new randomized controlled trials (RCTs) have been published after 2018. Therefore, this systematic review and meta-analysis compared survival rates between patients with nasopharyngeal carcinoma receiving IC-CCRT or concurrent chemoradiotherapy (CCRT).

**Methods:**

Four databases were searched for RCTs on this topic. Two authors independently selected studies, assessed evidence, and extracted data on progression-free survival, overall survival, metastasis-free survival, and local recurrence-free survival. Available data were pooled in a random-effects model and mainly presented in hazard ratio (HR). Heterogeneity and small study effects were also evaluated.

**Results:**

Eleven RCTs (n = 3345) were deemed eligible. Pooled results revealed that patients receiving IC-CCRT had significantly improved progression-free survival (HR = 0.66, *P* < 0.05), overall survival (HR = 0.64, *P* < 0.05), metastasis-free survival (HR = 0.58, *P* < 0.05), and local recurrence-free survival (HR = 0.69, *P* < 0.05) at 3 years, but no significant difference in 5-year overall survival was noted between IC-CCRT and CCRT (HR = 0.84, *P* > 0.05). Most findings had low heterogeneity.

**Conclusion:**

IC-CCRT may benefit patients with nasopharyngeal carcinoma in the medium term, although no significant difference was observed in 5-year survival compared with CCRT. All outcomes had decreased survival rate from the 3-years to 5-year follow-up. Differences in patient ethnicities and regimens of IC-CCRT may be sources of heterogeneity.

## Introduction

Nasopharyngeal carcinoma most commonly originates in the fossa of Rosenmüller, making it difficult to detect (1). According to Global Burden of Disease reports, nasopharyngeal carcinoma causes a decrease in lifespan by 13–15 years in terms of age-standardized years of life lost (2, 3). Peak incidence of nasopharyngeal carcinomas is middle age, and the male-to-female ratio is 3:1. However, because of the shortened lifespan of the middle-aged patient, even if cured, the quality of life is considerably affected.

Nasopharyngeal carcinoma is extremely common in the Asian population (4, 5), as well as in populations in North Africa, especially Tunisia and Algeria (6), and among Inuits in Alaska, North Canada, and Greenland (7). HLA subtypes A2, B14, and B46 are associated with an increased risk. The association of Epstein–Barr virus with nasopharyngeal carcinoma can be exploited in the diagnostic process, therapeutic strategies, and preventive treatment (8).

According to the 2018 National Comprehensive Cancer Network (NCCN) guidelines, the treatment algorithm for locoregionally advanced nasopharyngeal carcinoma (T1, N1–3; T2–4, any N) is concurrent chemoradiotherapy (CCRT) alone or followed by adjuvant chemotherapy or induction chemotherapy followed by CCRT. Induction chemotherapy can facilitate organ preservation, avoid morbid surgery, and improve the patient’s overall quality of life (9). The NCCN guidelines state that the intervention is appropriate based on any level of evidence in IC-CCRT in the advice of treatment of locoregionally advanced nasopharyngeal carcinoma; however, most of the relevant cited studies in the guidelines pertain to head and neck cancer.

Comparison of IC-CCRT and CCRT for nasopharyngeal carcinoma is worthy of further investigation due to incompleteness of evidence in previous syntheses although many head-to-head meta-analyses (10–16) and network meta-analyses have tried to provide conclusive evidence on this topic (17–24). Most studies have compared IC-CCRT with CCRT based on limited evidence, particularly the network meta-analyses. The most recent network meta-analyses were published in 2019 (22–24), whereas the largest network meta-analysis was published in 2017 (20). The most complete network meta-analysis included 27 trials, but direct evidence of IC-CCRT and CCRT in the network meta-analysis only relied on five trials (20). In fact, nine RCTs that had been published before the network meta-analysis was accepted for publication were not included in it (25–33).

In addition to the limited evidence in the previous syntheses, findings in the abovementioned studies are conflict with each other. For instance, one meta-analysis on the JAMA Network Open revealed that the addition of induction chemotherapy but not adjuvant chemotherapy to radiotherapy or CCRT can yield prolonged overall survival, progression-free survival, distance metastasis-free survival, and local recurrence-free survival (34). On the contrary, another meta-analysis on Journal of Clinical Oncology concluded that the addition of induction chemotherapy to CCRT does not present the highest survival benefit or consistent improvement for all end points (19). This inconclusive evidence may reduce clinicians’ confidence in applying the findings to clinical practice.

Thus, the benefit of IC-CCRT remains controversial, and most trials lack a sufficient sample size. Moreover, limited evidence has indicated the medium- and long-term benefits of IC-CCRT, and new randomized controlled trials (RCTs) have been published after 2018 (35–39). Therefore, this systematic review and meta-analysis included RCTs to compare survival rates between IC-CCRT and CCRT in treating nasopharyngeal carcinoma.

## Materials and methods

This systematic review and meta-analysis was conducted according to methodological guidance from the Cochrane handbook and Preferred Reporting Items for Systematic Reviews and Meta-analyses (PRISMA) reporting guideline (40, 41). According to the study aim stated earlier, our research question in PICO form is as follows:

P: Patients with nasopharyngeal carcinoma receiving concurrent chemoradiotherapy

I: Induction chemotherapy

C: No induction chemotherapy

O: Overall survival, disease-free survival, local recurrence-free survival, and metastasis-free survival

To evidence the efficacy of induction chemotherapy in patients with nasopharyngeal carcinoma undergoing concurrent chemoradiotherapy, this systematic review and meta-analysis only included RCTs. The inclusion criteria were as follows: (a) RCTs that recruited only patients with nasopharyngeal carcinoma and (b) all patients received CCRT. The present systematic review did not exclude studies based on treatment protocol, regimen, or dose of induction chemotherapy.

### Databases, search strategy, and study selection

We searched the Cochrane Central Register of Controlled Trials and Embase, New PubMed, and Web of Science databases for RCTs from inception until study date by using the following keywords in free-text and medical subject heading (MeSH): “nasopharyngeal carcinoma,” “chemoradiotherapy,” “chemotherapy,” “radiotherapy,” and “induction”. Next, the keywords were combined using Boolean operators “OR” and “AND.” Synonyms were connected by “OR,” and keyword sets of different concepts were connected by “AND” (**Appendix 1**). No filter was used for language and publication date. Reference lists of relevant systematic reviews and RCTs on this topic were also screened for retrieving potential evidence.

Two authors (TCH and CJC) independently reviewed titles and abstracts of the identified references; after exclusion of duplicated and irrelevant references, full-texts were retrieved for further review and eligibility judgment. Any disagreement was resolved through discussion with an experienced researcher.

### Data extraction and quality assessment

TCH and CJC then independently extracted and double-checked information on study area; inclusion duration; sex; age; proportion of advanced stage; regimen, dose, and schedule of chemotherapy; protocol and dose of radiation therapy; study design; and overall, disease-free, local recurrence-free, and metastasis-free survival. Data on outcomes of interest were usually reported as HR (with CI) or events; if both were present, the authors extracted both.

The authors then evaluated the quality of the included RCTs byusing the Cochrane risk of bias tool (41). Generation of randomization, allocation concealment, blinding to investigators, blinding to participants, blinding to assessors, loss to follow-up, type of analysis, and selective reporting were evaluated. In case of disagreements, an experienced author (corresponding author) made the final decision.

### Analysis and statistics

Qualitative synthesis was performed through tabulation with relevant discussion, and quantitative synthesis was conducted using R 4.0.2 for Microsoft Windows. Log HR with standard error (SE) for outcomes of interest were derived from the extracted HR and CI. Primary outcomes were mainly based on pooled log HR with SE. Data on survival cases and total sample size were pooled for revealing risk ratio (RR) for each year after treatment because the included trials reported results at various time points. Both pooled HR and RR were estimated using the random-effects model due to heterogeneity in not only statistical findings but also clinical conditions. Statistical heterogeneity was tested with *I*² and *P* value for the heterogeneity test: values of >50% and <0.10, respectively, indicated high heterogeneity. Graphical display of study heterogeneity (GOSH) analysis was conducted using the “gosh” function for objects of class “rma” in library “metafor,” and a funnel plot was generated to assess potential publication bias.

## Results

**Figure 1:**
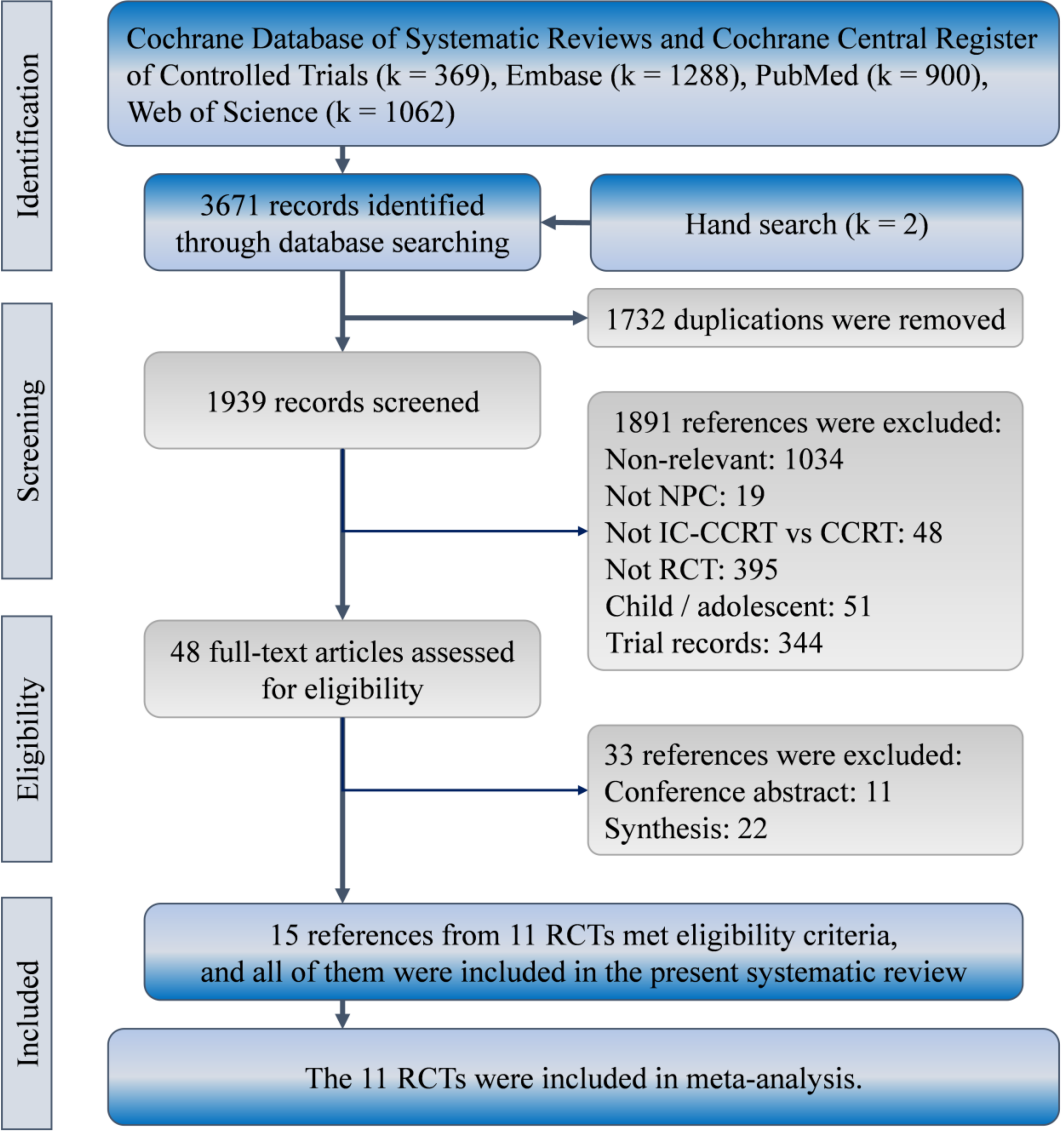
Patient selection flowchart according to PRISMA guidelines. IC-CCRT, induction chemotherapy; RCT, randomized controlled trial.

We identified 3671 (2844) references in the initial search. Eventually, 15 references presented results of 11 RCTs comparing IC-CCRT with CCRT alone for management of nasopharyngeal carcinoma (25–29, 31–33, 35–39, 42, 43), and were considered for the present synthesis (**Figure 1**).

### Characteristics and quality of included studies

A total of 3345 patients with nasopharyngeal carcinoma were recruited in the 11 RCTs for qualitative and quantitative synthesis. They participated in clinical trials between 2002 and 2016. Most patients were from Asia (n = 3123, 93%), and only 222 (7%) were from Europe. More than half of them were men (n = 2449, 73%), and the median age was between 42 and 51.6 years. More than 99% patients had advanced-stage carcinoma but no metastasis **Table 1**. Other information on regimen, dose, and treatment protocol is presented in **Supplementary Table 1**, and the quality of the included RCTs is indicated in **Supplementary Table 2**. Some concerns of risk of bias were raised due to the unclear bias of risk in allocation concealment. Biases from performance, detection, attrition, and selected reporting were low in most trials, except in the trial by Jin et al. (33).

**Table 1:** Characteristics of the included randomized controlled trials.

		Inclusion	Sex/	(M/F)	Median	age	<stage IV/	stage IV, n	Chemo	regimen
Author	Location	Years	IC-CCRT	CCRT	IC-CCRT	CCRT	IC-CCRT	CCRT	IC-CCRT	CCRT
Li et al., 2019 (37)/Sun et al., 2016 (31)/Li et al., 2016 (30)/Zhang et al., 2018 (42)	China	2011–2013	193/48	174/65	42	44	129/112	133/106	Docetaxel Cisplatin 5-fluorouracil	Cisplatin
Zhang et al., 2019 (43)	China	2013–2016	182/60	164/74	46	45	111/131	120/118	Gemcitabine Cisplatin	Cisplatin
Yang et al., 2019 (38)/Cao et al., 2017 (32)	China	2008–2015	173/65	190/48	44	42	118/120	133/105	Cisplatin Fluorouracil	Cisplatin
Frikha et al., 2018 (35)	France, Tunisia	2009–2012	28/12	32/9	46 ^a^	48 ^a^	N/A	N/A	Docetaxel Cisplatin 5-fluorouracil	Cisplatin
Hong et al., 2018 (36)	Taiwan	2003–2009	176/63	179/61	45	47	0/239	0/240	Mitomycin Epirubicin Cisplatin 5-fluorouracil Leucovorin	Cisplatin
Jin et al., 2017 (33)	China	2009–2012	227/69	255/88	Overall:	46	224/72	272/71	Cisplatin 5-fluorouracil	Cisplatin
Tan et al., 2015 (29)	Singapore	2004–2012	71/15	63/23	48.5	51.6	50/36	53/33	Paclitaxel Carboplatin Gemcitabine	Cisplatin
Gao et al., 2013 (28)	China	2008–2009	43/14	39/16	Overall:	18–60	14/43	11/44	Cisplatin 5-fluorouracil	Cisplatin
Fountzilas et al., 2012 (26)	Greece, Romania	2003–2008	51/21	48/21	49	51	41/31	42/27	Epirubucin Paclitaxil Cisplatin	Cisplatin
Huang et al., 2012 (27)	China	2003–2006	56/44	60/40	43.7 ^a^	45.2 ^a^	67/33	56/44	Carboplatin 5-fluorouracil	Carboplatin
Hui et al.,2009 (25)	Hong Kong	2002–2004	21/13	24/7	50	45	19/15	19/12	Docetaxel Cisplatin	Cisplatin

a mean; M/F, male/female; NR, no report.

### Progression-free survival

**Figure 2:**
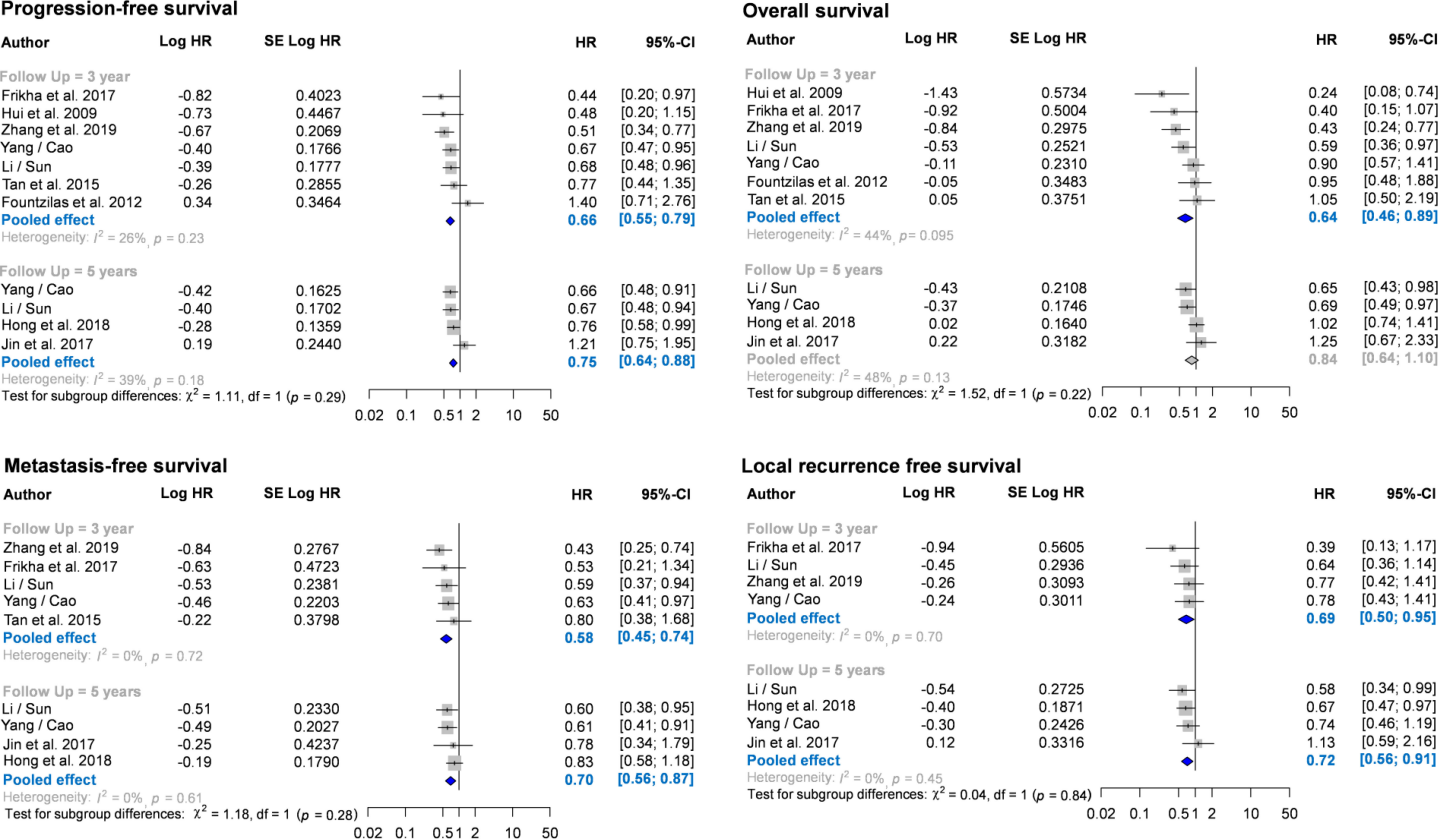
Forest plots for meta-analysis of hazard ratio of survival. CI, confidence interval; HR, hazard ratio.

Nine RCTs (n = 3013) reported HR and CI for disease-free survival. Seven of them reported 3-year disease-free survival (25, 26, 29, 32, 35, 37–39), and four reported 5-year disease-free survival (**Figure 2**) (33, 36–38). Compared with the CCRT group, the IC-CCRT group had lower HR in 3-year disease-free survival (HR = 0.66, 95% CI: 0.55–0.79; I^2^ = 26%, *P* for heterogeneity > 0.10) and lower HR in 5-year disease-free survival (HR = 0.75, 95% CI: 0.64–0.88; I^2^ = 39%, *P* for heterogeneity > 0.10). Small study effects may not seriously affect this finding.

**Figure 3:**
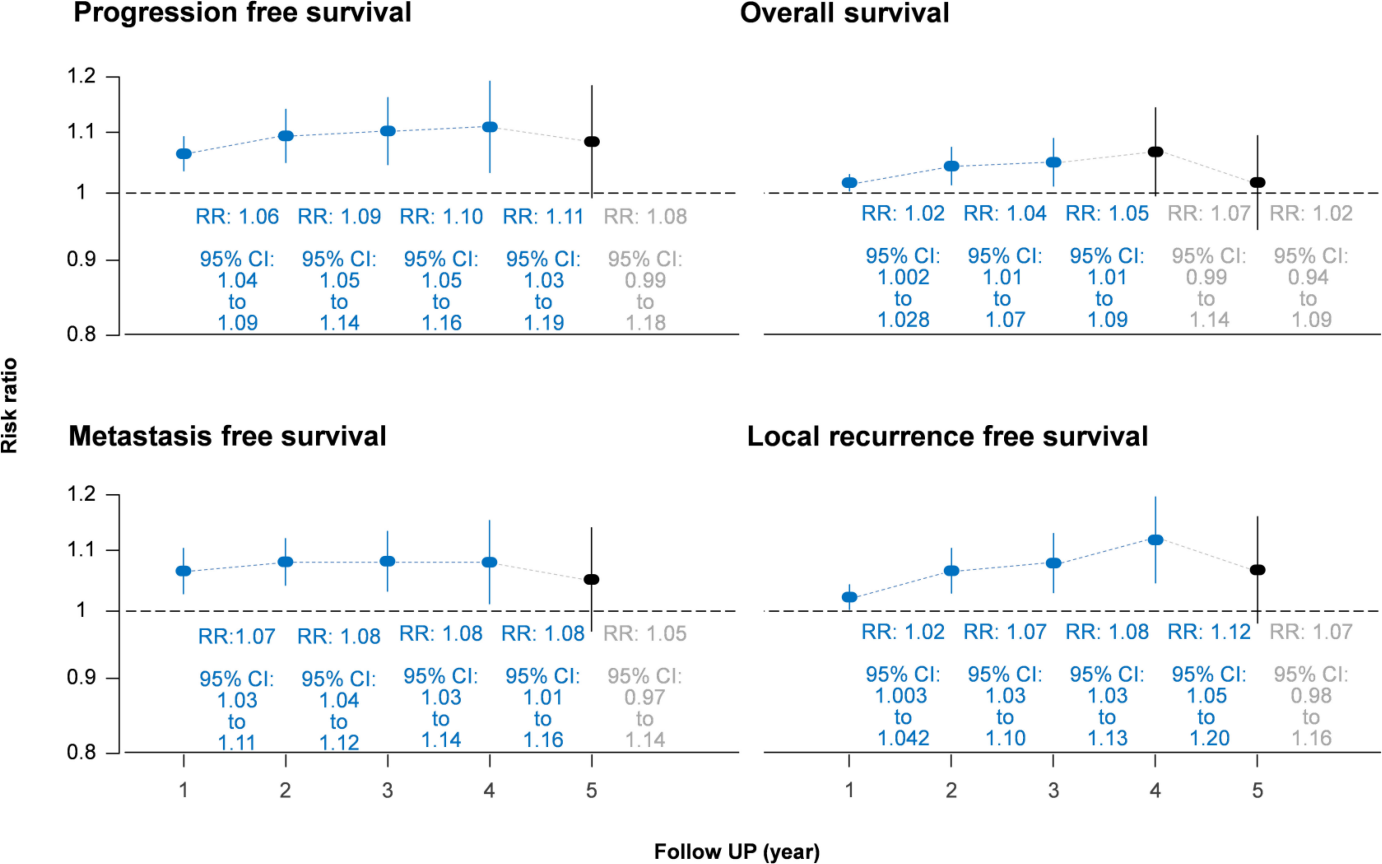
Forest plot of cumulative survival rate using risk ratio. CI, confidence interval; RR, risk ratio.

**Figure 4:**
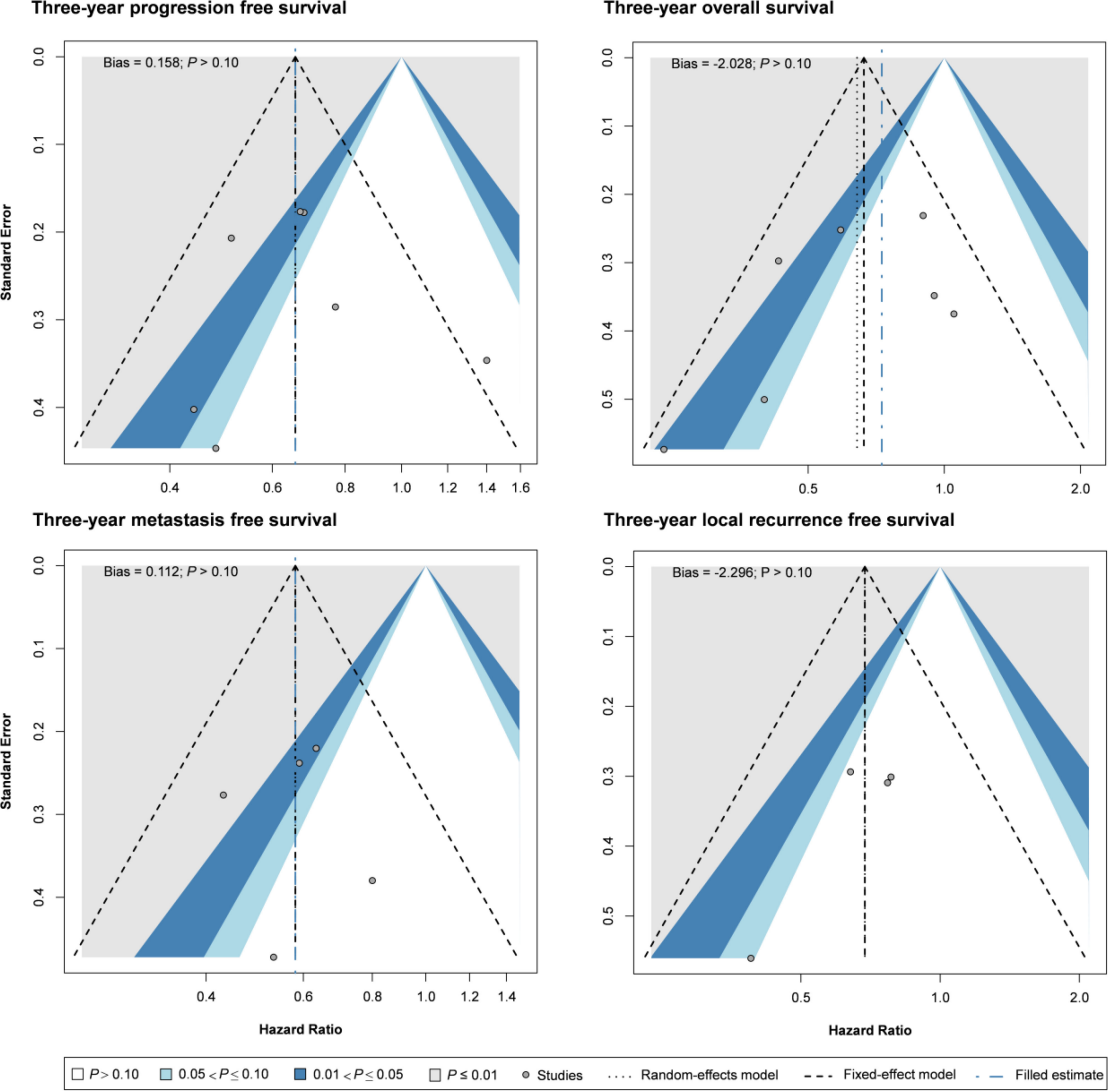
Funnel plots for meta-analysis of hazard ratio of survival. Bias, Egger’s regression intercept for publication bias test.

The IC-CCRT group had a significantly higher cumulative disease-free survival rate than the CCRT group at 1 year (RR = 1.06, 95% CI: 1.04–1.09; I^2^ = 32%, *P* for heterogeneity > 0.10), 2 years (RR = 1.09, 95% CI: 1.05–1.14; I^2^ = 1%, *P* for heterogeneity > 0.10), 3 years (RR = 1.10, 95% CI: 1.05–1.16; I^2^ = 0%, *P* for heterogeneity > 0.10), and 4 years (RR = 1.11, 95% CI: 1.03–1.19; I^2^ = 0%, *P* for heterogeneity > 0.10) but not at 5 years (I^2^ = 36%, *P* for heterogeneity > 0.10; **Figure 3**; **Appendix 2**). Small study effects may not seriously affect disease-free survival (**Figure 4**; **Appendix 3**).

### Overall survival rate

The nine RCTs (n = 3013) also reported HR and CI for overall survival. Seven of them reported 3-year overall survival (25, 26, 29, 32, 35, 37–39), and four reported 5-year overall survival (33, 36–38). The pooled results revealed that IC-CCRT significantly improved 3-year overall survival (HR = 0.64, 95% CI: 0.46–0.89; I^2^ = 44%, *P* for heterogeneity > 0.05), but no significant intergroup difference was observed in 5-year overall survival (I^2^ = 48%, *P* for heterogeneity > 0.10). Small study effects may not seriously affect 3-year overall survival.

In the measurement of cumulative rate, the overall survival rate in the IC-CCRT group was significantly higher than that in the CCRT group at 1 year (RR = 1.02, 95% CI: 1.002–1.028; I^2^ = 39%, *P* for heterogeneity < 0.10), 2 years (RR = 1.04, 95% CI: 1.01–1.07; I^2^ = 47%, *P* for heterogeneity < 0.10), and 3 years (RR = 1.05, 95% CI: 1.01–1.09; I^2^ = 0%, *P* for heterogeneity > 0.10; **Appendix 4**) but not at 4 and 5 years. Small study effects may not seriously affect overall survival (**Appendix 5**).

### Metastasis-free survival

Seven RCTs (n = 2807) also reported HR and CI for metastasis-free survival. Of them, five reported 3-year metastasis-free survival (29, 32, 35, 37–39), and four reported 5-year metastasis-free survival (33, 36–38). Pooled estimates revealed that IC-CCRT resulted in a significantly better metastasis-free survival than CCRT did at 3 years (HR = 0.58, 95% CI: 0.45–0.73; I^2^ = 0%, *P* for heterogeneity > 0.10) and 5 years (HR = 0.70, 95% CI: 0.56–0.87; I^2^ = 0%, *P* for heterogeneity > 0.10). Small study effects may not seriously affect this finding.

Cumulative metastasis-free survival rate in the IC-CCRT group was significantly higher than that in the CCRT group at 1 year (RR = 1.07, 95% CI: 1.03–1.11; I^2^ = 48%, *P* for heterogeneity > 0.05), 2 years (RR = 1.08, 95% CI: 1.04–1.12; I^2^ = 0%, *P* for heterogeneity > 0.10), 3 years (RR = 1.08, 95% CI: 1.03–1.14; I^2^ = 0%, *P* for heterogeneity > 0.10), and 4 years (RR = 1.08, 95% CI: 1.01–1.16; I^2^ = 0%, *P* for heterogeneity > 0.10) but not at 5 years (I^2^ = 28%, *P* for heterogeneity > 0.10; **Appendix 6**). Small study effects may not seriously affect cumulative metastasis-free survival (**Appendix 7**).

### Local recurrence-free survival

Six RCTs (n = 2506) also reported HR and CI for local recurrence-free survival. Of them, four reported 3-year local recurrence-free survival (32, 35, 37–39), and four reported 5-year local recurrence-free survival (33, 36–38). Pooled estimates indicated that IC-CCRT exhibited a significant improvement in local recurrence-free survival in both 3-year follow-up (HR = 0.69, 95% CI: 0.50–0.95; I^2^ = 0%, *P* for heterogeneity > 0.10) and 5-years follow-up (HR = 0.72, 95% CI: 0.56–0.91; I^2^ = 0%, *P* for heterogeneity > 0.10). Small study effects may not seriously affect the pooled estimate of local recurrence-free survival.

**Figure 5:**
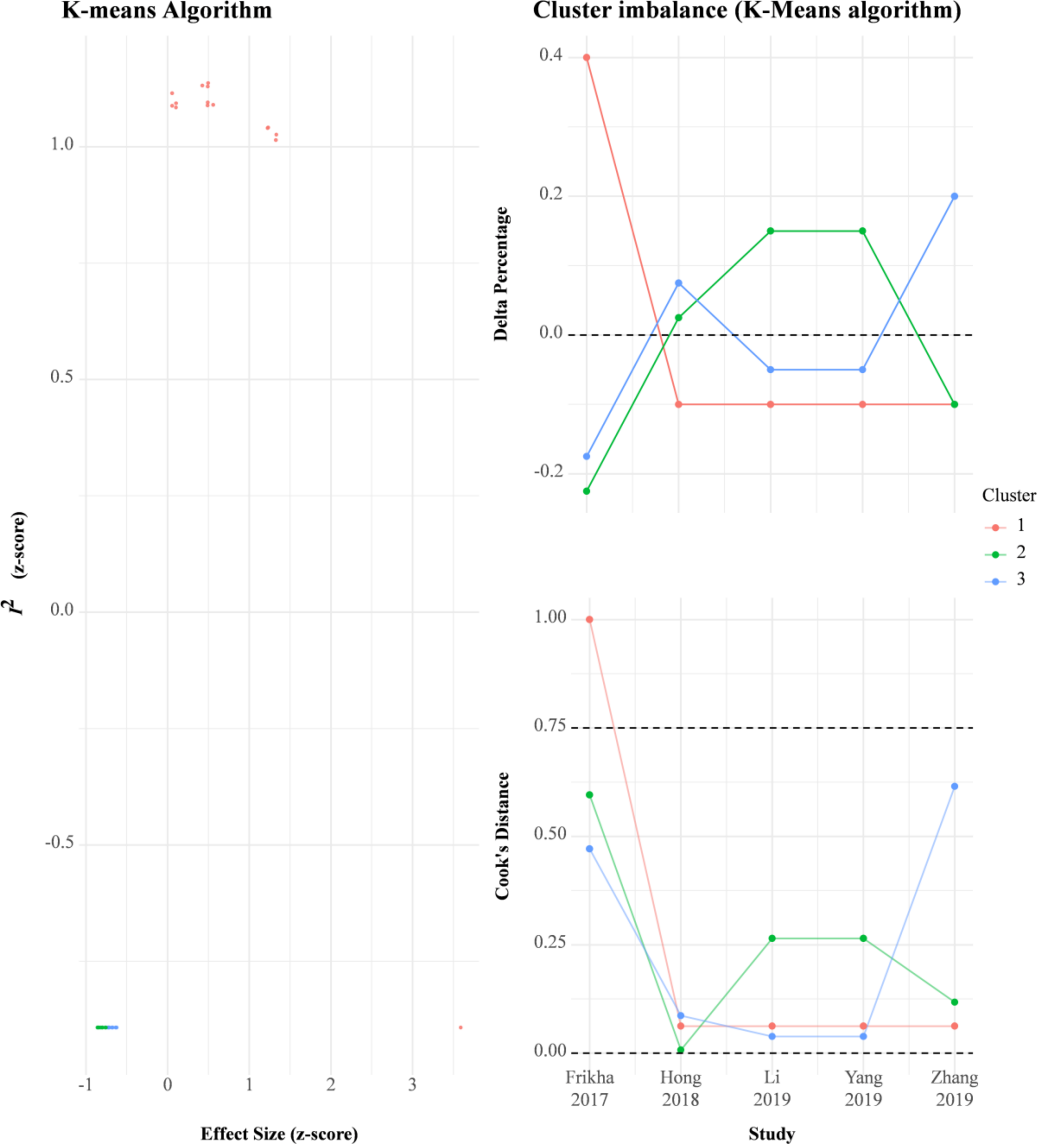
GOSH plots for meta-analysis of cumulative Local recurrence-free survival rate.

Similarly, the local recurrence-free survival rate in the IC-CCRT group was significantly higher than that in the CCRT group at 1 year (RR = 1.02, 95% CI: 1.003–1.042; I^2^ = 63%, *P* for heterogeneity < 0.05), 2 years (RR = 1.07, 95% CI: 1.03–1.10; I^2^ = 54%, *P* for heterogeneity > 0.05), 3 years (RR = 1.08, 95% CI: 1.03–1.13; I^2^ = 0%, *P* for heterogeneity > 0.10), and 4 years (RR = 1.12, 95% CI: 1.05–1.20; I^2^ = 0%, *P* for heterogeneity > 0.10) but not at 5 years (I^2^ = 7%, *P* for heterogeneity > 0.10; **Appendix 8**). GOSH analysis revealed that heterogeneity could be reduced by excluding the study by Frikha et al. (**Figure 5** and **Appendix 9**). Small study effects may not seriously affect cumulative local recurrence-free survival (**Appendix 10**).

## Discussion

### Key findings

The results of our meta-analysis demonstrated significant improvement in medium-term (3-year) overall survival, progression-free survival, local-regional free survival, and metastasis-free survival in patients with advanced-stage nasopharyngeal carcinoma who receive induction chemotherapy additional to CCRT, whereas no such benefit was seen in long-term (5-year) overall survival, as evidenced by the trend observed in both HR and cumulative measurements. Moreover, some heterogeneity was observed in overall survival, cumulative measurements for metastasis-free survival at 1 year, and cumulative measurements for local recurrence-free survival at 1 and 2 years. Briefly, both time-to-event and cumulative measurements exhibit favorable trends toward IC-CCRT although the two measurements are inconsistent in statistical significance of 5-year progression-free survival, local-regional free survival, and metastasis-free survival. Overall, current evidence on relevant outcomes of 3-year survival is of moderate certainty, whereas evidence on 5-year survival outcomes has low to very low certainty (**Supplementary Table 3**).

The heterogeneity might be due to different ethnicities, patients’ baseline condition, or regimens of the induction chemotherapy. First, two studies were conducted in nonendemic areas (one in Tunisia and France and the other in Greece and Romania) (26, 35), and the outcomes were relatively poor. However, the association between human ethnicities and disease outcome remains unconfirmed due to the complexity of race and variations in regimens of the induction chemotherapies. Although no significant difference in overall survival and progression-free survival was noted among the induction chemotherapy of TPF, PF, and TP (22), some trials did not use common regimens of TPF, PF, and TP for induction chemotherapy (26, 29, 39). The regimen of induction chemotherapies in most of the included studies comprised two or all of docetaxel, cisplatin, and 5-flurouracine, except studies by Zhang et al. (gemcitabine and cisplatin) (39), Tan et al. (paclitaxel, gemcitabine, and carboplatin) (29), and Fountzilas et al. (epirubicin, paclitaxel, and cisplatin) (26). These differences may have led to heterogeneous estimates in data pooling, which was also indicated by GOSH analysis.

To be specific, poorer outcomes of progression-free survival and overall survival were indicated in the studies by Fountzilas et al. and Tan et al. (26, 29) and may have been due to more undesirable adverse effects caused by different regimens. In the trial by Fountzilas et al., for instance, more than 10% of cases in the IC-CCRT group discontinued due to toxicity (n = 2), withdrawal (n = 4), or no reason (n = 1) (26). Despite our finding that induction chemotherapy leads to better medium-term outcome, potential risks of adverse effects due to additional chemotherapy and poorer quality of life during therapy should be considered during clinical decision-making.

Compared with trials by Fountzilas et al. and Tan et al., better survival outcomes could be found in the study by Zhang et al., (26, 29, 39) probably because it included fewer patients with N2 and N3 stage disease who have a higher risk of disease progression (39). Unfortunately, data are insufficient to analyze the interaction between the N stage and the effects of induction chemotherapy in patients with nasopharyngeal carcinoma. Future studies should attempt to evaluate this association. Furthermore, identification of an optimal strategy of IC-CCRT may help in the management of local nasopharyngeal carcinoma. The optimal combination strategies and indications for using induction chemotherapy warrant further evaluation.

### Previous syntheses

Many relevant syntheses have been published in this decade, and meta-analysis of RCTs is worthy of a further discussion. Reduced certainty by methodological and statistical heterogeneity weakens the confidence of pooled results in meta-analyses using data from observational studies although those meta-analyses have more cases than the meta-analyses only using data from RCTs. For instance, a head-to-head meta-analysis by Tan et al. included 11 studies with 2802 cases (29), but only 6 were RCTs (25, 26, 29, 31, 32, 35). Inclusion of non-RCTs led to seemingly uncertain and unreliable pooled results due to highly heterogeneity (I^2^ = 62%) (12). Besides, our synthesis is also based on RCTs. In consequence, it would be appropriate to compare findings in the present synthesis with those in the meta-analyses using data from RCTs.

Our findings are consistent with recent meta-analyses of RCTs (13, 15, 16, 22). Meta-analysis by Wang et al. seems to be the largest synthesis of RCTs comparing IC-CCRT and CCRT for nasopharyngeal carcinoma amongst relevant syntheses on this topic (10–24), and it is a head-to-head meta-analysis of 10 RCTs with 2280 individuals (16). IC-CCRT appears to be an effective strategy for treating nasopharyngeal carcinoma since it improves progression-free survival (Peto’s odds ratio [POR] = 0.75, 95% CI: 0.65–0.87) and overall survival (POR = 0.7, 95% CI: 0.56–0.87) and based on cumulative measurement (16). However, their study used cumulative measurement only without separation of time points. Knowledge regarding time factors or trends in the relevant outcomes of nasopharyngeal carcinoma management is essential for clinicians. Time-to-event analysis is more informative than cumulative measurement with unclear time frames. Based on time-to-event measurement, IC-CCRT also significantly improves 3-year failure-free survival (HR = 0.67, 95% CI: 0.55–0.80), 5-year failure-free survival (HR = 0.70, 95% CI: 0.58–0.83), 3-year overall survival (HR = 0.70, 95% CI: 0.55–0.89), and 5-year overall survival (HR = 0.77, 95% CI: 0.62–0.94) (15). Nevertheless, these findings are only based on seven RCTs.

In addition to the abovementioned meta-analyses, a review of meta-analyses is also worthy of further discussion since it concludes oppositely after taking many meta-analyses on relevant topics into consideration (44). On the basis of the works of five earlier meta-analyses (10–12, 17, 45), the review indicates no concrete evidence in favor of routine addition of induction chemotherapy to CCRT in managing patients with locoregionally advanced nasopharynx cancer. Actually, we agree with their concerns because the earlier meta-analyses seem to have no effects of IC-CCRT on overall survival with relatively small sample size. Indeed, non-significant finding in 5-year overall survival might decrease patients’ willingness in receiving IC-CCRT; wherefore, CCRT alone might be still a good option for some patients.

The present meta-analysis complements the understanding of the effects of addition of induction chemotherapy to CCRT in nasopharyngeal carcinoma management. Our findings may be more reliable because we included 11 RCTs with more than 3000 cases in total. Primary outcomes, progression-free survival, and overall survival were not seriously affected by heterogeneity or small study effects. The quality and completeness of our findings are much higher than those of previous studies. Moreover, our study provides a clearer overview of the outcomes because we evaluated both time-to-event and cumulative measurements with separate time points. This study further provides a summary of findings according to the Grading of Recommendations Assessment, Development and Evaluation (GRADE) approach (**Supplementary Table 3**), and may thus improve knowledge translation from academic research to clinical practice.

For clinical practice, quality of life after both IC-CCRT and CCRT is critical point and may be worthy of further discussion although there is limited evidence on quality of life between these two treatment strategies for nasopharyngeal carcinoma (46). Induction chemotherapy appears to result in better quality of life as compared with those without induction chemotherapy, and the finding may be due to few toxicities in patients with nasopharyngeal carcinoma after IC-CCRT (46, 47). However, another study indicates that patient receiving IC-CCRT may have lower quality-adjusted life year and disability-adjusted life year than those receiving CCRT (48). Because quality of life might affect decision-making, further studies are warranted to investigate the relevant outcomes between IC-CCRT and CCRT.

### Limitations

This study has several limitations. First, most patients had advanced nasopharyngeal carcinoma, precluding the drawing of firm evidence for those with early-stage disease. More evidence is also required for the application of IC-CCRT to non-Asian ethnicities with nasopharyngeal carcinoma because >90% of the participants in this study were from Asia. GOSH analysis also indicated that heterogeneity was caused by a study from Europe. Second, differences in the regimens for induction chemotherapy were noted, but evidence consistently supported IC-CCRT. Clinical heterogeneity of the induction chemotherapy regimen is a threat to the internal validity of this synthesis. Future studies should investigate which regimen for IC-CCRT achieves the best outcomes.

## Conclusions

According to the available data from RCTs, the present meta-analysis indicated that IC-CCRT may benefit patients with nasopharyngeal carcinoma, although no significant difference in 5-year survival was noted between IC-CCRT and CCRT. Due to the non-significance, clinicians might need to reconsider before the uses of IC-CCRT, or to have a shared-decision making for this situation. There are trends toward no differences in all measured outcomes between IC-CCRT and CCRTat the 5-year follow-up; however, some heterogeneity may exist due to differences in ethnicities and regimens. These sources of heterogeneity warrant further research.

## Data availability statement

The original contributions presented in the study are included in the article/**Supplementary Material**. Further inquiries can be directed to the corresponding authors.

## Authors contributions

Conceptualization: Y-FD. and Y-NK. Data curation: T-CH and C-JC. Formal analysis: C-JC and Y-NK. Investigation: T-CH, C-JC, and Y-FD. Methodology: Y-NK. Interpretation: T-CH, C-JCand Y-FD. Supervision: Y-FD. Visualization: Y-NK. Writing – original draft: T-CH and C-JC. Writing – review & editing: Y-FD and Y-NK. All authors contributed to the article and approved the submitted version.

## Funding

This study received grant from Wan Fang Hospital, Taipei Medical University with grant number 111-wf-phd-04.

## Conflict of interest

The authors declare that the research was conducted in the absence of any commercial or financial relationships that could be construed as a potential conflict of interest.

## Publisher’s note

All claims expressed in this article are solely those of the authors and do not necessarily represent those of their affiliated organizations, or those of the publisher, the editors and the reviewers. Any product that may be evaluated in this article, or claim that may be made by its manufacturer, is not guaranteed or endorsed by the publisher.

## Abbreviations

CCRT, concurrent chemoradiotherapy; CI, confidence interval; IC, induction chemotherapy; GOSH, Graphical display of study heterogeneity; HR, hazard ratio; RCT, randomized controlled trial; RR, risk ratio; WMD, weighted mean difference.
