# Supplementary material for: Impact of induction chemotherapy with concurrent chemoradiotherapy on nasopharyngeal carcinoma: A meta-analysis of randomized controlled trials
Source: Front Oncol. 2022 Sep 13;12:965719. doi: 10.3389/fonc.2022.965719 (PMC9513799; doi:10.3389/fonc.2022.965719)

Frontiers in Oncology

Impact of induction chemotherapy with concurrent chemoradiotherapy on nasopharyngeal carcinoma:

a meta-analysis of randomized controlled trials

(Appendices)

Authors:

Ting-Chieh Huang, M.D.[1], Chi-Jen Chen, M.D.[1], Yi-Fang Ding, M.D.[1], and Yi-No Kang, Consultant, M.A. [2,3,4,5]

Affiliations:

1. Department of Otorhinolaryngology, Wan Fang Hospital, Taipei Medical University, Taipei, Taiwan
2. Evidence-Based Medicine Center, Wan Fang Hospital, Taipei Medical University, Taipei, Taiwan
3. Research Center of Big Data and Meta-analysis Center, Wan Fang Hospital, Taipei Medical University, Taipei, Taiwan
4. Cochrane Taiwan, Taipei Medical University
5. Institute of Health Policy & Management, College of Public Health, Taipei, Taiwan

Supplementary table 1

Details of the included trials

Trial	Group	Chemo Regimen	Chemo Dose	Chemo Schedule	RT Protocol	RT Dose
Li 2019/<br>Sun 2016/<br>Li 2016/<br>Zhang 2018	I-CCRT	Docetaxel<br>Cisplatin<br>5-FU	75 mg/m^2 /D1<br>75 mg/m^2 /D1<br>600 mg/m^2 /D 1–5	3-week *<br>3cycles	IMRT<br>68–70 Gy in 2.0–2.27 Gy per fraction with five daily fractions per week for 6–7 weeks; a moderate dose escalation to ≤74 Gy or dose increase per fraction to ≤2.35 Gy could be considered for some patients	≥ 66 Gy to the primary tumor and ≥ 50 Gy to bilateral cervical lymph nodes and potential sites of local infiltration
	CCRT	Cisplatin	100 mg/m^2	Q3W on D1, 22, and 43		
Zhang 2019	I-CCRT	Gemcitabine<br>Cisplatin	1000mg/m^2 /D1, D8<br>80mg/m^2 /D1	3-week *<br>3cycles	IMRT	Unknown
	CCRT	Cisplatin	100 mg/m^2	Q3W on D1, 22, and 43		
Yang 2019/<br>Cao 2017	I-CCRT	Cisplatin<br>Fluorouracil	80 mg/m^2 /D1<br>800 mg/m^2 /D1-5	3-week * 2 cycles	two-dimensional radiotherapy (2DRT) or intensity-modulated radiotherapy (IMRT)	external beam RT daily fractions a week at 2.0-2.33 Gy per fraction
	CCRT	Cisplatin	80 mg/m^2	Q3W on D1, 22, and 43		
Frikha 2018	I-CCRT	Docetaxel<br>Cisplatin<br>5-FU	75 mg/m^2 /D1<br>75 mg/m^2 /D1<br>750 mg/m^2 /D1-5	3-week *<br>3cycles	Unknown	70 Gy in 2 Gy/fraction, 5 fractions per week with an overall time of 7 weeks
	CCRT	Cisplatin	40 mg/m^2	QW * 7weeks		
Hong 2018	I-CCRT	Mitomycin<br>Epirubicin<br>Cisplatin<br>5-FU<br>Leucovorin	8 mg/m^2 /D1<br>60 mg/m^2 /D1<br>60 mg/m^2 /D1<br>450 mg/m^2 /D8<br>30 mg/m^2 /D8	3-week * 3 cycles	140/240 (58%) CCRT arm 152/239 (64%) I-CCRT arm received IMRT	1.8–2.2 Gy per fraction with 5 daily fractions QW, total ≥ 70 Gy: primary tumor, 66–70 Gy: involved neck
	CCRT	Cisplatin	30 mg/m^2	start on first RT<br>QW		
Jin 2017	I-CCRT	Cisplatin<br>5-fluorouracil	80-100 mg/m^2 /D1<br>800-1000 mg/m^2 /D1-5	3-week * 2-3 cycles	Unclear	Primary tumor66 to 72 Gy at 2.12, to 2.27 Gy/fraction, involved LNs 62 to 70 Gy, high-risk clinical target volume 60 Gy, and low-risk clinical target volume 54 Gy
	CCRT	Cisplatin	80-100 mg/m^2 /D1	Q3W * 3 cycles		
Tan 2015	I-CCRT	Paclitaxel<br>Carboplatin<br><br>Gemcitabine	70 mg/m^2 /D1, D8<br>Dosed to target area under the concentration-time curve of 2.5 infused over 1 hour /D1, D8<br>1000mg/m^2 / D1, D8	3-week * 3 cycles	Two-dimensional radiotherapy (2DRT) or intensity-modulated radiotherapy (IMRT)	2DRT: Primary tumor + LN 70 Gy in 35 2-Gy fractions. The rest :60 Gy in 30 fractions.<br>IMRT: Gross disease with margin 69.96 Gy in 33*2.12-Gy fractions, the rest 60 Gy in 1.82-Gy fractions
	CCRT	Cisplatin	40 mg/m^2	QW * 8 cycles		
Gao 2013	I-CCRT	DDP<br>5-FU	30mg/m^2 /D1-3<br>450mg/m^2 /D1-3	every 3 weeks for 2 cycles	Unclear	2 Gy per fraction, 5 fractions per week
	CCRT		40mg/m^2	QW		
Fountzilas 2012	I-CCRT	Epirubicin -><br>Paclitaxil -><br>Cisplatin	75 mg/m^2 /D1<br>175 mg/m^2 /D1<br>75 mg/m^2 /D2	3-week * 3 cycles	External beam RT by conventional fractionation	66–70 Gy (in 6.5–7 weeks): primary tumor, 66 Gy: clinically involved LN <3 cm, 70 Gy: LN ≥ 3 cm, 50 Gy: to uninvolved cervical and supraclavicular areas
	CCRT	Cisplatin	40 mg/m^2 1hr before RT	QW		
Huang 2012	I-CCRT	Carboplatin<br>5-FU	AUC=6<br>75 mg/m^2 /D1-5	One week	Unclear	2 Gy per day for 5 times a week
	CCRT	Carboplatin	AUC=6 /D7, 28, 49			
Hui 2009	I-CCRT	Docetaxel<br>Cisplatin	75 mg/m^2 /D1<br>75 mg/m^2 /D1	3-week * 2 cycles	Ho's technique	78.4 Gy (±8.6 Gy) in ICCRT and 76.5 Gy (±7.4 Gy) in CCRT
	CCRT	Cisplatin	40 mg/m^2	QW*8		

Supplementary table 2

Risk of bias

Trial	Randomization	Concealment	Blinding	Follow-up	Analysis type	Selective report
Li 2019/ Sun 2016/ Li 2016/ Zhang 2018	Low	Low	Low	Low	ITT	Low
Zhang 2019	Low	Unclear	Low	Low	ITT and PP	Low
Yang 2019/ Cao 2017	Low	Low	Low	Low	ITT	Low
Frikha 2018	Low	Low	Low	Low	ITT	Low
Hong 2018	Low	Unclear	Low	Low	ITT	Low
Jin 2017	Unclear	Unclear	Low	Low	Unknown	Low
Tan 2015	Low	Unclear	Low	Low	ITT	Low
Gao 2013	Unclear	Unclear	Low	Low	ITT	Low
Fountzilas 2012	Low	Unclear	Low	Low	ITT	Unclear
Huang 2012	Unclear	Unclear	Low	Low	ITT	Unclear
Hui 2009	Low	Unclear	Low	Low	ITT	Low

Supplementary table 3

Summary of findings

No. of participants (studies)	Certainty of the Evidence (GRADE)	Relative effects (95% CI)	Comments
**3-year progression-free survival**			
1415 (7 RCTs)	⊕⊕⊕◯ [a] MODERATE	**HR 0.66** (0.55 to 0.79)	IC-CCRT increases the 3-year progression-free survival rate.
**5-year progression-free survival**			
1598 (4 RCTs)	⊕⊕◯◯ [a,b] LOW	**HR 0.75** (0.64 to 0.88)	IC-CCRT increases the 5-year progression-free survival rate.
**3-year overall survival**			
1415 (7 RCTs)	⊕⊕⊕◯ [a] MODERATE	**HR 0.64** (0.46 to 0.89)	IC-CCRT increases the 3-year overall survival rate.
**5-year overall survival**			
1598 (4 RCTs)	⊕◯◯◯ [a,b,c] VERY LOW	**HR 0.84** (0.64 to 1.30)	IC-CCRT does not increase the 5-year overall survival rate.
**3-year metastasis-free survival**			
1209 (5 RCTs)	⊕⊕⊕◯ [a] MODERATE	**HR 0.58** (0.45 to 0.74)	IC-CCRT increases the 3-year metastasis-free survival rate.
**5-year metastasis-free survival**			
1598 (4 RCTs)	⊕⊕◯◯ [a,b] LOW	**HR 0.70** (0.56 to 0.87)	IC-CCRT increases the 5-year metastasis-free survival rate.
**3-year local recurrence-free survival**			
1037 (4 RCTs)	⊕⊕⊕◯ [a] MODERATE	**HR 0.69** (0.50 to 0.95)	IC-CCRT increases the 3-year local recurrence-free survival rate.
**5-year local recurrence-free survival**			
1598 (4 RCTs)	⊕⊕◯◯ [a,b] LOW	**HR 0.72** (0.56 to 0.91)	IC-CCRT increases the 3-year local recurrence-free survival rate.

HR, hazard ratio; IC-CCRT, induction chemotherapy combined with concurrent chemoradiotherapy; RCT, randomized controlled trial

[a], Downgraded a level due to relatively small sample size (n < 2000).

[b], Downgraded a level because many items of risk of bias were unclear in the included trials.

[c], Downgraded a level due to obvious heterogeneity (I^2 > 50% or P for heterogeneity < 0.10).

Appendix 1

Search strategy (Primary search strategy and syntax in Cochrane database)

Primary search strategy

#1. Nasopharynx cancer
#2. Nasopharynx tumor
#3. Nasopharynx carcinoma
#4. Nasopharyngeal cancer
#5. Nasopharyngeal tumor
#6. Nasopharyngeal carcinoma
#7. Nasopharyngeal neoplasm
#8. Nasopharynx neoplasm
#9. #1 OR #2 OR #3 OR #4 OR #5 OR #6 OR #7 OR #8
#10. induction chemotherapy
#11. neoadjuvant chemotherapy
#12. #10 OR #11
#13. #9 AND #12

Syntax in Cochrane Database of Systematic Reviews and Cochrane Central Register of Controlled Trials :

(Nasopharynx cancer OR Nasopharynx tumor OR Nasopharynx carcinoma OR Nasopharyngeal cancer OR Nasopharyngeal tumor OR Nasopharyngeal carcinoma OR Nasopharyngeal neoplasm OR Nasopharynx neoplasm) AND (induction chemotherapy OR neoadjuvant chemotherapy)

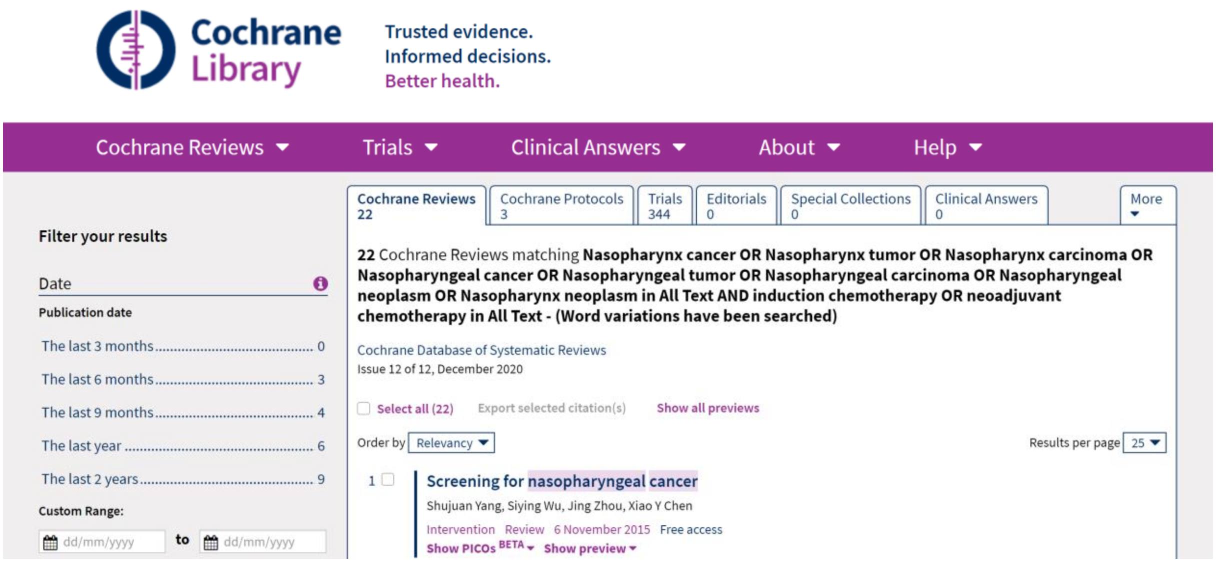

Appendix 1
Search strategy (Embase)

Syntax in Embase:

('cancer, nasopharynx'/exp OR 'cancer, nasopharynx' OR 'epipharynx cancer'/exp OR 'epipharynx cancer' OR 'nasopharyngeal cancer'/exp OR 'nasopharyngeal cancer' OR 'nasopharynx cancer'/exp OR 'nasopharynx cancer' OR 'rhinopharyngioma'/exp OR 'rhinopharyngioma' OR 'rhinopharynx cancer'/exp OR 'rhinopharynx cancer' OR 'epipharynx tumor'/exp OR 'epipharynx tumor' OR 'epipharynx tumour'/exp OR 'epipharynx tumour' OR 'nasopharyngeal neoplasms'/exp OR 'nasopharyngeal neoplasms' OR 'nasopharyngeal tumor'/exp OR 'nasopharyngeal tumor' OR 'nasopharyngeal tumour'/exp OR 'nasopharyngeal tumour' OR 'nasopharynx tumor'/exp OR 'nasopharynx tumor' OR 'nasopharynx tumour'/exp OR 'nasopharynx tumour' OR 'rhinopharynx tumor'/exp OR 'rhinopharynx tumor' OR 'rhinopharynx tumour'/exp OR 'rhinopharynx tumour' OR 'epipharynx carcinoma'/exp OR 'epipharynx carcinoma' OR 'nasopharyngeal carcinoma'/exp OR 'nasopharyngeal carcinoma' OR 'nasopharynx carcinoma'/exp OR 'nasopharynx carcinoma' OR 'postnasal space carcinoma'/exp OR 'postnasal space carcinoma' OR 'rhinopharyngeal carcinoma'/exp OR 'rhinopharyngeal carcinoma' OR 'rhinopharynx carcinoma'/exp OR 'rhinopharynx carcinoma') AND ('neoadjuvant chemotherapy'/exp OR 'neoadjuvant chemotherapy' OR 'chemotherapy, induction'/exp OR 'chemotherapy, induction' OR 'induction chemotherapy'/exp OR 'induction chemotherapy')

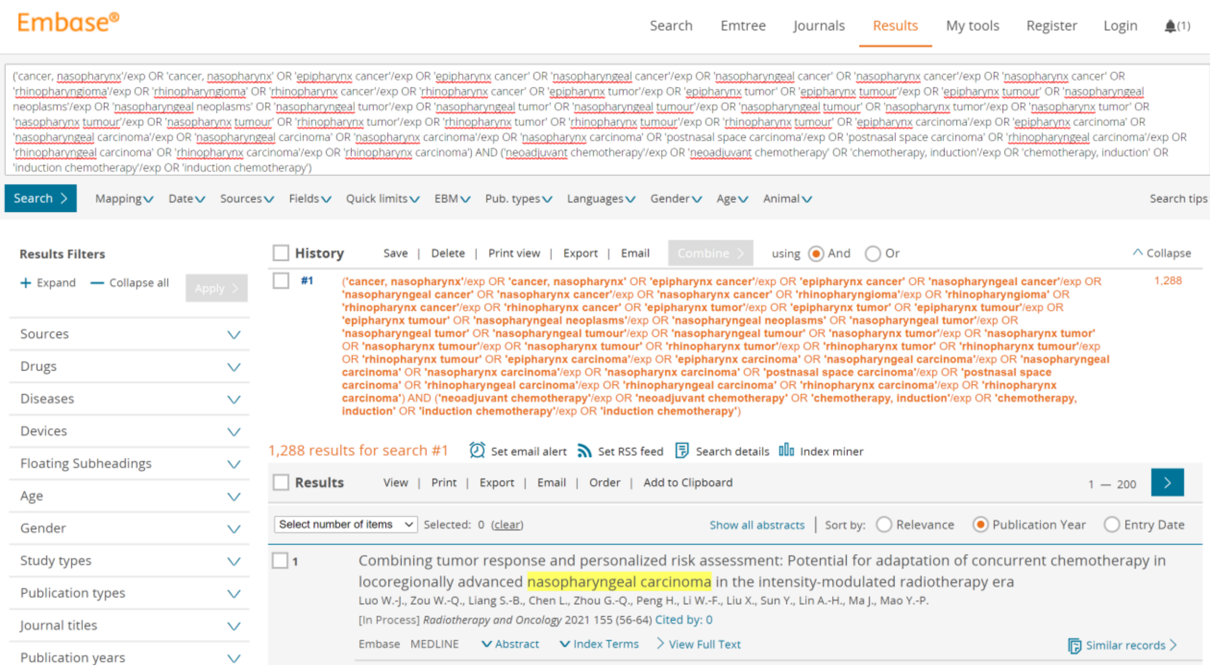

Appendix 1
Search strategy (PubMed)

Syntax in PubMed:

("nasopharyngeal neoplasms"[MeSH Terms] OR ("nasopharyngeal"[All Fields] AND "neoplasms"[All Fields]) OR "nasopharyngeal neoplasms"[All Fields] OR ("nasopharynx"[All Fields] AND "cancer"[All Fields]) OR "nasopharynx cancer"[All Fields] OR ("nasopharyngeal neoplasms"[MeSH Terms] OR ("nasopharyngeal"[All Fields] AND "neoplasms"[All Fields]) OR "nasopharyngeal neoplasms"[All Fields] OR ("nasopharynx"[All Fields] AND "tumor"[All Fields]) OR "nasopharynx tumor"[All Fields]) OR ("nasopharyngeal carcinoma"[MeSH Terms] OR ("nasopharyngeal"[All Fields] AND "carcinoma"[All Fields]) OR "nasopharyngeal carcinoma"[All Fields] OR ("nasopharynx"[All Fields] AND "carcinoma"[All Fields]) OR "nasopharynx carcinoma"[All Fields]) OR ("nasopharyngeal neoplasms"[MeSH Terms] OR ("nasopharyngeal"[All Fields] AND "neoplasms"[All Fields]) OR "nasopharyngeal neoplasms"[All Fields] OR ("nasopharyngeal"[All Fields] AND "cancer"[All Fields]) OR "nasopharyngeal cancer"[All Fields] OR "nasopharyngeal carcinoma"[MeSH Terms] OR ("nasopharyngeal"[All Fields] AND "carcinoma"[All Fields]) OR "nasopharyngeal carcinoma"[All Fields] OR ("nasopharyngeal"[All Fields] AND "cancer"[All Fields])) OR ("nasopharyngeal neoplasms"[MeSH Terms] OR ("nasopharyngeal"[All Fields] AND "neoplasms"[All Fields]) OR "nasopharyngeal neoplasms"[All Fields] OR ("nasopharyngeal"[All Fields] AND "tumor"[All Fields]) OR "nasopharyngeal tumor"[All Fields]) OR ("nasopharyngeal carcinoma"[MeSH Terms] OR ("nasopharyngeal"[All Fields] AND "carcinoma"[All Fields]) OR "nasopharyngeal carcinoma"[All Fields]) OR ("nasopharyngeal neoplasms"[MeSH Terms] OR ("nasopharyngeal"[All Fields] AND "neoplasms"[All Fields]) OR "nasopharyngeal neoplasms"[All Fields] OR ("nasopharyngeal"[All Fields] AND "neoplasm"[All Fields]) OR "nasopharyngeal neoplasm"[All Fields]) OR ("nasopharyngeal neoplasms"[MeSH Terms] OR ("nasopharyngeal"[All Fields] AND "neoplasms"[All Fields]) OR "nasopharyngeal neoplasms"[All Fields] OR ("nasopharynx"[All Fields] AND "neoplasm"[All Fields]) OR "nasopharynx neoplasm"[All Fields])) AND ("induction chemotherapy"[MeSH Terms] OR ("induction"[All Fields] AND "chemotherapy"[All Fields]) OR "induction chemotherapy"[All Fields] OR (("neoadjuvancy"[All Fields] OR "neoadjuvant therapy"[MeSH Terms] OR ("neoadjuvant"[All Fields] AND "therapy"[All Fields]) OR "neoadjuvant therapy"[All Fields] OR "neoadjuvant"[All Fields] OR "neoadjuvants"[All Fields] OR "neoadjuvent"[All Fields]) AND ("chemotherapy s"[All Fields] OR "drug therapy"[MeSH Terms] OR ("drug"[All Fields] AND "therapy"[All Fields]) OR "drug therapy"[All Fields] OR "chemotherapies"[All Fields] OR "drug therapy"[MeSH Subheading] OR "chemotherapy"[All Fields])))

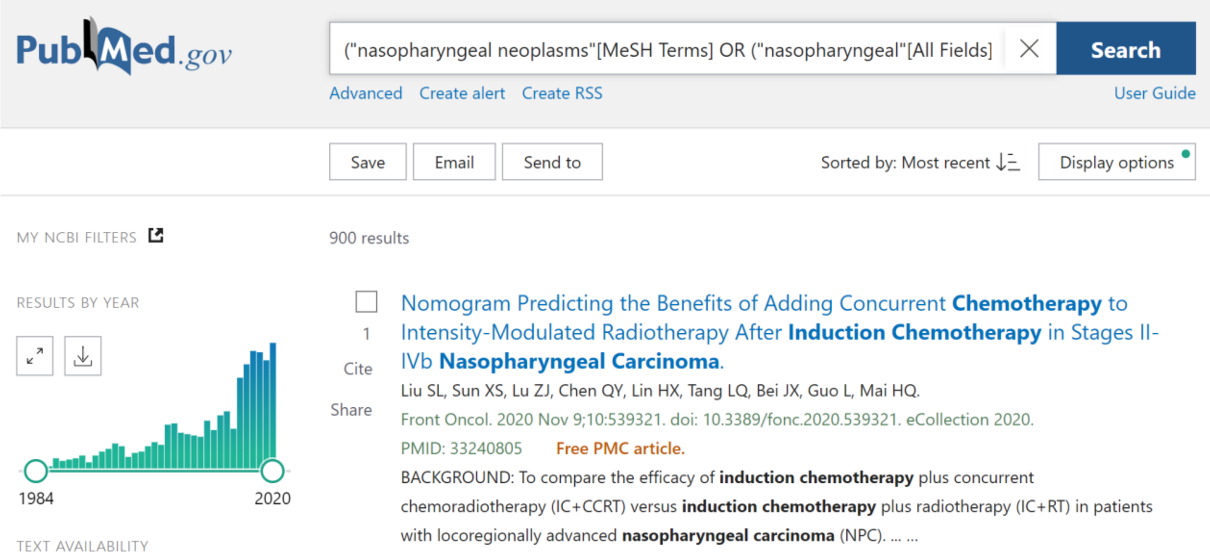

Appendix 1
Search strategy (Web of Science)

Syntax in Web of Science :

TOPIC: (Nasopharynx cancer OR Nasopharynx tumor OR Nasopharynx carcinoma OR Nasopharyngeal cancer OR Nasopharyngeal tumor OR Nasopharyngeal carcinoma OR Nasopharyngeal neoplasm OR Nasopharynx neoplasm) AND TOPIC: (induction chemotherapy OR neoadjuvant chemotherapy)

Timespan: All years. Indexes: SCI-EXPANDED, SSCI, A&HCI, ESCI.

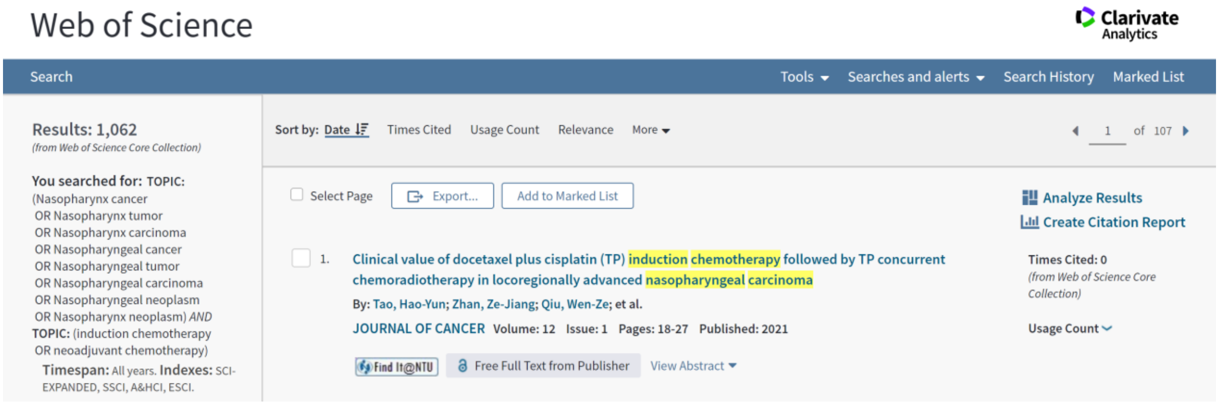

Appendix 2 to 10

Findings of meta-analysis, small study effects, and GOSH analysis

Appendix 2

Forest plot for progression-free survival

Study	CCRT Events	CCRT Total	IC-CCRT Events	IC-CCRT Total	RR	95%-CI
**FollowUp = 1**						
Yang et al. 2019	218	238	213	238	1.02	[0.97; 1.08]
Zhang et al. 2019	234	242	217	238	1.06	[1.01; 1.11]
Li et al. 2019	225	241	210	239	1.06	[1.00; 1.13]
Hong et al. 2018	212	239	200	240	1.06	[0.99; 1.14]
Tan et al. 2015	84	86	73	86	1.15	[1.05; 1.27]
Frikha et al. 2017	38	40	31	41	1.26	[1.04; 1.52]
**Pooled effect**		.		.	**1.06**	**[1.04; 1.09]**
Heterogeneity: I^2 = 32%, p = 0.20						
**FollowUp = 2**						
Yang et al. 2019	206	238	193	238	1.07	[0.99; 1.16]
Hong et al. 2018	183	239	170	240	1.08	[0.97; 1.20]
Zhang et al. 2019	215	242	194	238	1.09	[1.01; 1.18]
Tan et al. 2015	57	86	52	86	1.10	[0.87; 1.38]
Li et al. 2019	203	241	181	239	1.11	[1.02; 1.22]
Frikha et al. 2017	34	40	23	41	1.52	[1.12; 2.05]
**Pooled effect**		.		.	**1.09**	**[1.05; 1.14]**
Heterogeneity: I^2 = 1%, p = 0.41						
**FollowUp = 3**						
Tan et al. 2015	32	86	32	86	1.00	[0.68; 1.47]
Huang et al. 2012	70	100	68	100	1.03	[0.85; 1.24]
Gao et al. 2013	36	57	32	55	1.09	[0.80; 1.46]
Yang et al. 2019	193	238	176	238	1.10	[1.00; 1.21]
Zhang et al. 2019	146	242	130	238	1.10	[0.95; 1.29]
Li et al. 2019	191	241	170	239	1.11	[1.00; 1.24]
Hong et al. 2018	175	239	155	240	1.13	[1.00; 1.28]
Frikha et al. 2017	22	40	19	41	1.19	[0.77; 1.83]
**Pooled effect**		.		.	**1.10**	**[1.05; 1.16]**
Heterogeneity: I^2 = 0%, p = 0.99						
**FollowUp = 4**						
Frikha et al. 2017	6	40	7	41	0.88	[0.32; 2.39]
Tan et al. 2015	20	86	21	86	0.95	[0.56; 1.63]
Hong et al. 2018	141	239	130	240	1.09	[0.93; 1.27]
Yang et al. 2019	162	238	148	238	1.09	[0.96; 1.25]
Li et al. 2019	181	241	161	239	1.11	[0.99; 1.25]
Zhang et al. 2019	93	242	73	238	1.25	[0.98; 1.61]
**Pooled effect**		.		.	**1.11**	**[1.03; 1.19]**
Heterogeneity: I^2 = 0%, p = 0.91						
**FollowUp = 5**						
Frikha et al. 2017	1	40	5	41	0.21	[0.03; 1.68]
Tan et al. 2015	7	86	16	86	0.44	[0.19; 1.01]
Yang et al. 2019	135	238	127	238	1.06	[0.90; 1.25]
Li et al. 2019	169	241	152	239	1.10	[0.97; 1.25]
Hong et al. 2018	107	239	97	240	1.11	[0.90; 1.36]
Zhang et al. 2019	35	242	26	238	1.32	[0.82; 2.13]
**Pooled effect**		.		.	**1.08**	**[0.99; 1.18]**
Heterogeneity: I^2 = 36%, p = 0.17						

Test for subgroup differences: χ^2 = 2.72, df = 4 (p = 0.61)

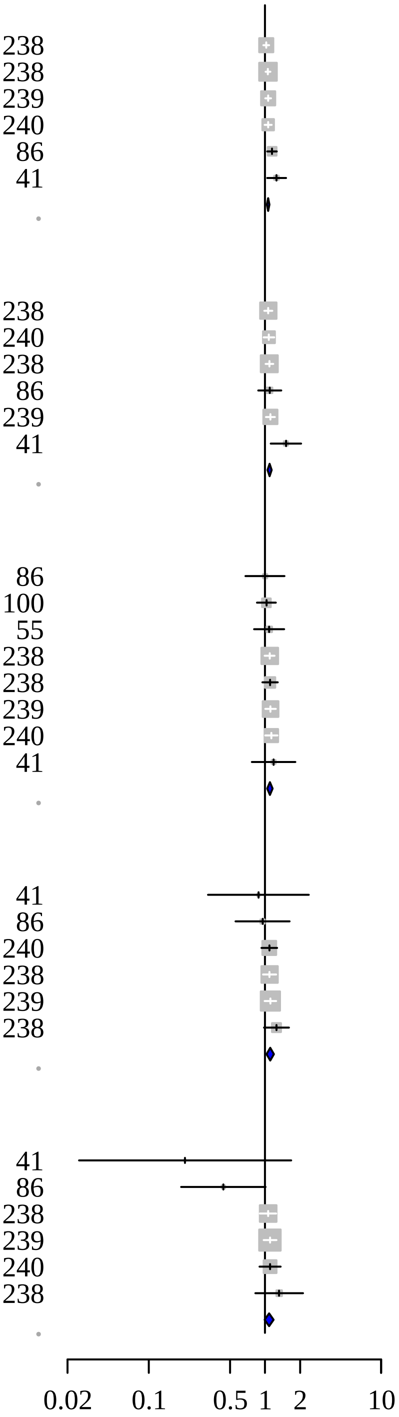

Appendix 3

Small study effects in three-year progression-free survival

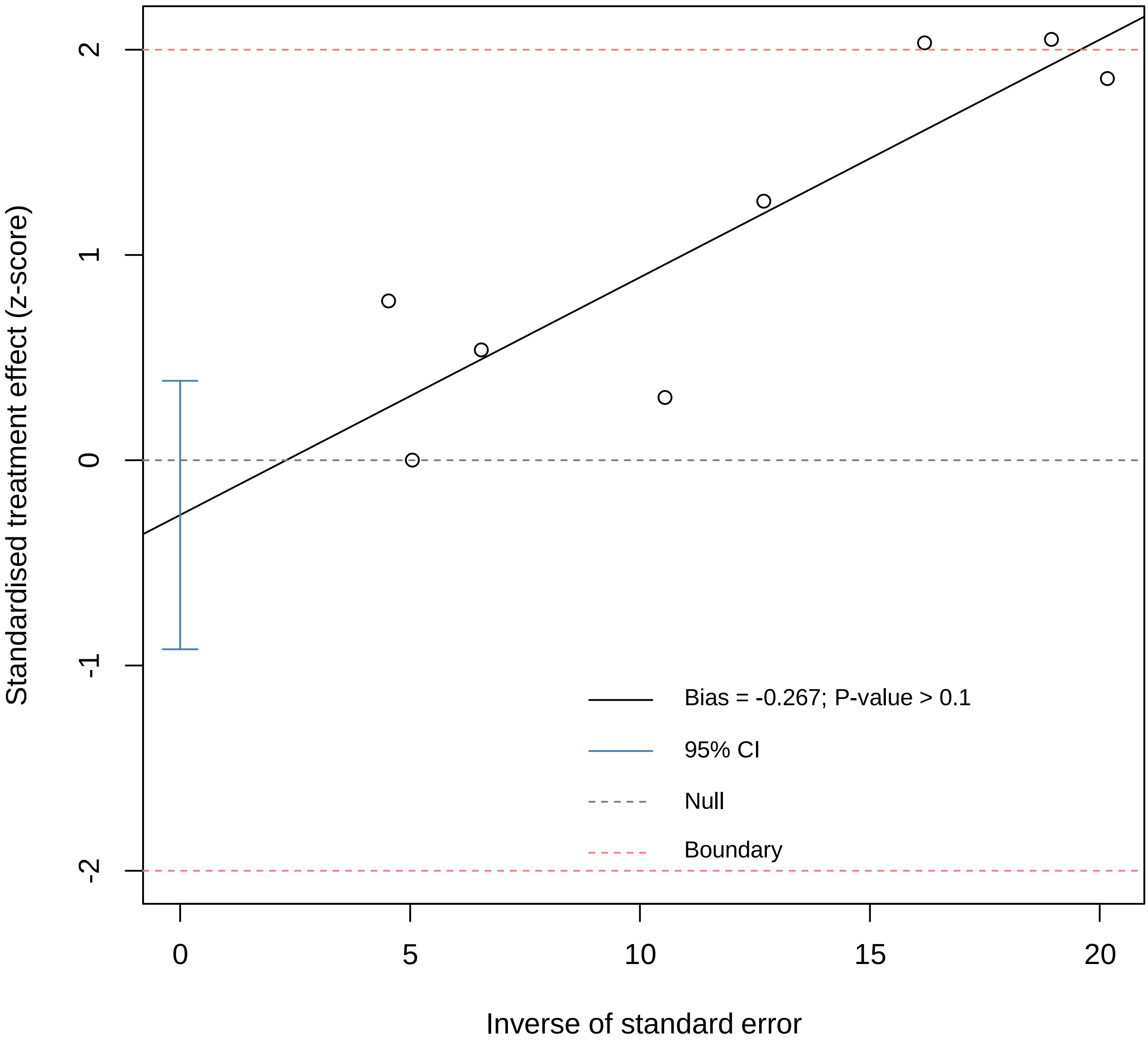

Appendix 4

Forest plot for overall survival

Study	CCRT Events	CCRT Total	IC-CCRT Events	IC-CCRT Total	RR	95%-CI
**FollowUp = 1**						
Yang et al. 2019	233	238	233	238	1.00	[0.97; 1.03]
Li et al. 2019	234	241	231	239	1.00	[0.97; 1.04]
Hong et al. 2018	226	239	223	240	1.02	[0.97; 1.07]
Zhang et al. 2019	241	242	232	238	1.02	[1.00; 1.04]
Tan et al. 2015	86	86	84	86	1.02	[0.99; 1.06]
Frikha et al. 2017	40	40	34	41	1.20	[1.05; 1.38]
**Pooled effect**		.		.	**1.02**	**[1.00; 1.03]**
Heterogeneity I^2 = 39%, p = 0.15						
**FollowUp = 2**						
Li et al. 2019	223	241	221	239	1.00	[0.95; 1.05]
Yang et al. 2019	224	238	218	238	1.03	[0.98; 1.08]
Hong et al. 2018	212	239	201	240	1.06	[0.99; 1.14]
Zhang et al. 2019	236	242	219	238	1.06	[1.02; 1.11]
Tan et al. 2015	66	86	61	86	1.08	[0.91; 1.29]
Frikha et al. 2017	38	40	29	41	1.34	[1.09; 1.66]
**Pooled effect**		.		.	**1.04**	**[1.01; 1.07]**
Heterogeneity I^2 = 47%, p = 0.10						
**FollowUp = 3**						
Tan et al. 2015	41	86	43	86	0.95	[0.70; 1.29]
Yang et al. 2019	211	238	208	238	1.01	[0.95; 1.08]
Li et al. 2019	216	241	205	239	1.04	[0.98; 1.12]
Zhang et al. 2019	162	242	152	238	1.05	[0.92; 1.19]
Gao et al. 2013	46	57	42	55	1.06	[0.87; 1.28]
Huang et al. 2012	84	100	79	100	1.06	[0.93; 1.21]
Hong et al. 2018	197	239	177	240	1.12	[1.02; 1.23]
Frikha et al. 2017	27	40	23	41	1.20	[0.85; 1.70]
**Pooled effect**		.		.	**1.05**	**[1.01; 1.09]**
Heterogeneity I^2 = 0%, p = 0.81						
**FollowUp = 4**						
Frikha et al. 2017	9	40	10	41	0.92	[0.42; 2.03]
Tan et al. 2015	26	86	28	86	0.93	[0.60; 1.45]
Yang et al. 2019	177	238	180	238	0.98	[0.89; 1.09]
Li et al. 2019	207	241	187	239	1.10	[1.01; 1.19]
Zhang et al. 2019	100	242	87	238	1.13	[0.90; 1.42]
Hong et al. 2018	160	239	142	240	1.13	[0.99; 1.30]
**Pooled effect**		.		.	**1.07**	**[0.99; 1.14]**
Heterogeneity I^2 = 0%, p = 0.51						
**FollowUp = 5**						
Frikha et al. 2017	2	40	8	41	0.26	[0.06; 1.13]
Tan et al. 2015	10	86	18	86	0.56	[0.27; 1.13]
Yang et al. 2019	148	238	152	238	0.97	[0.85; 1.12]
Li et al. 2019	186	241	178	239	1.04	[0.94; 1.15]
Hong et al. 2018	115	239	109	240	1.06	[0.88; 1.28]
Zhang et al. 2019	36	242	29	238	1.22	[0.77; 1.92]
**Pooled effect**		.		.	**1.02**	**[0.94; 1.09]**
Heterogeneity I^2 = 32%, p = 0.19						

Test for subgroup differences: χ^2 = 5.95, df = 4 (p = 0.20)

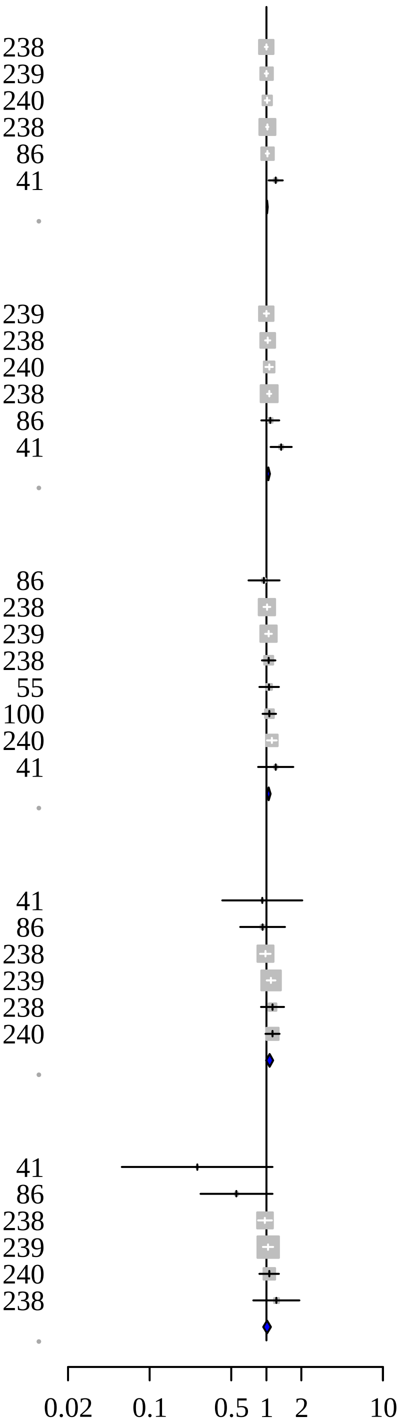

Appendix 5
Small study effects in three-year overall survival

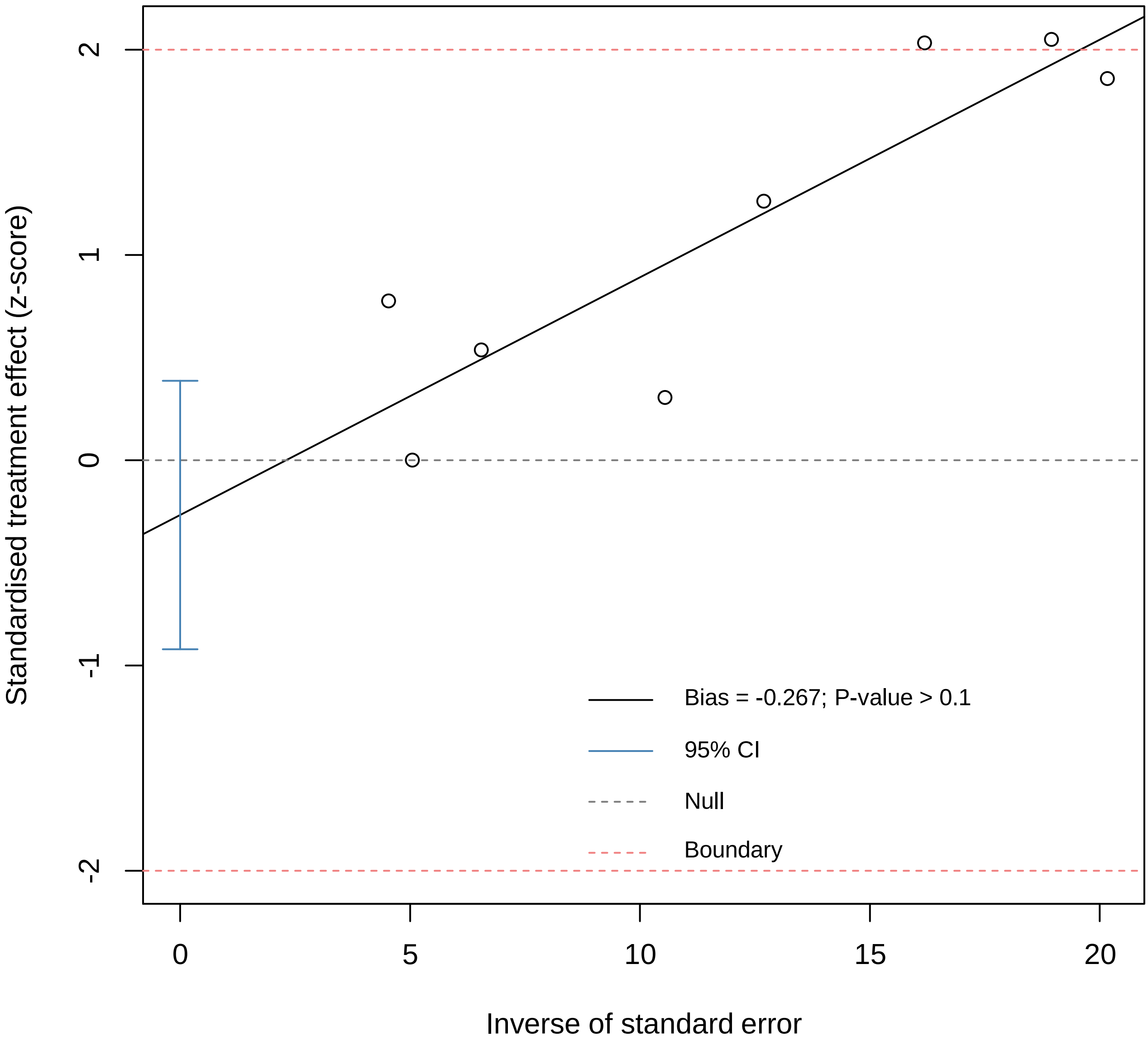

Appendix 6

Forest plot for metastasis-free survival

Study	CCRT Events	CCRT Total	IC-CCRT Events	IC-CCRT Total	RR	95%-CI
**FollowUp = 1**						
Yang et al. 2019	220	238	218	238	1.01	[0.96; 1.06]
Li et al. 2019	228	241	215	239	1.05	[1.00; 1.11]
Hong et al. 2018	212	239	200	240	1.06	[0.99; 1.14]
Zhang et al. 2019	238	242	217	238	1.08	[1.03; 1.13]
Tan et al. 2015	84	86	73	86	1.15	[1.05; 1.27]
Frikha et al. 2017	39	40	33	41	1.21	[1.03; 1.42]
**Pooled effect**		.		.	**1.07**	**[1.03; 1.11]**
Heterogeneity: $I^2 = 48\%$, $p = 0.09$						
**FollowUp = 2**						
Yang et al. 2019	209	238	200	238	1.04	[0.97; 1.12]
Li et al. 2019	214	241	197	239	1.08	[1.00; 1.16]
Hong et al. 2018	183	239	170	240	1.08	[0.97; 1.20]
Zhang et al. 2019	226	242	204	238	1.09	[1.02; 1.16]
Tan et al. 2015	57	86	52	86	1.10	[0.87; 1.38]
Frikha et al. 2017	34	40	25	41	1.39	[1.06; 1.84]
**Pooled effect**		.		.	**1.08**	**[1.04; 1.12]**
Heterogeneity: $I^2 = 0\%$, $p = 0.53$						
**FollowUp = 3**						
Tan et al. 2015	32	86	32	86	1.00	[0.68; 1.47]
Huang et al. 2012	81	100	78	100	1.04	[0.90; 1.20]
Yang et al. 2019	198	238	186	238	1.06	[0.97; 1.16]
Zhang et al. 2019	154	242	140	238	1.08	[0.94; 1.25]
Li et al. 2019	204	241	185	239	1.09	[1.00; 1.19]
Gao et al. 2013	39	57	34	55	1.11	[0.84; 1.45]
Hong et al. 2018	175	239	155	240	1.13	[1.00; 1.28]
Frikha et al. 2017	24	40	20	41	1.23	[0.82; 1.84]
**Pooled effect**		.		.	**1.08**	**[1.03; 1.14]**
Heterogeneity: $I^2 = 0\%$, $p = 0.98$						
**FollowUp = 4**						
Frikha et al. 2017	7	40	8	41	0.90	[0.36; 2.24]
Tan et al. 2015	20	86	21	86	0.95	[0.56; 1.63]
Yang et al. 2019	168	238	160	238	1.05	[0.93; 1.18]
Hong et al. 2018	141	239	130	240	1.09	[0.93; 1.27]
Li et al. 2019	194	241	176	239	1.09	[0.99; 1.21]
Zhang et al. 2019	96	242	80	238	1.18	[0.93; 1.50]
**Pooled effect**		.		.	**1.08**	**[1.01; 1.16]**
Heterogeneity: $I^2 = 0\%$, $p = 0.95$						
**FollowUp = 5**						
Frikha et al. 2017	2	40	6	41	0.34	[0.07; 1.59]
Tan et al. 2015	7	86	16	86	0.44	[0.19; 1.01]
Yang et al. 2019	140	238	134	238	1.04	[0.90; 1.22]
Li et al. 2019	179	241	168	239	1.06	[0.95; 1.18]
Hong et al. 2018	107	239	97	240	1.11	[0.90; 1.36]
Zhang et al. 2019	35	242	28	238	1.23	[0.77; 1.95]
**Pooled effect**		.		.	**1.05**	**[0.97; 1.14]**
Heterogeneity: $I^2 = 28\%$, $p = 0.22$						

Test for subgroup differences: $\chi^2 = 0.59$, df = 4 ($p = 0.96$)

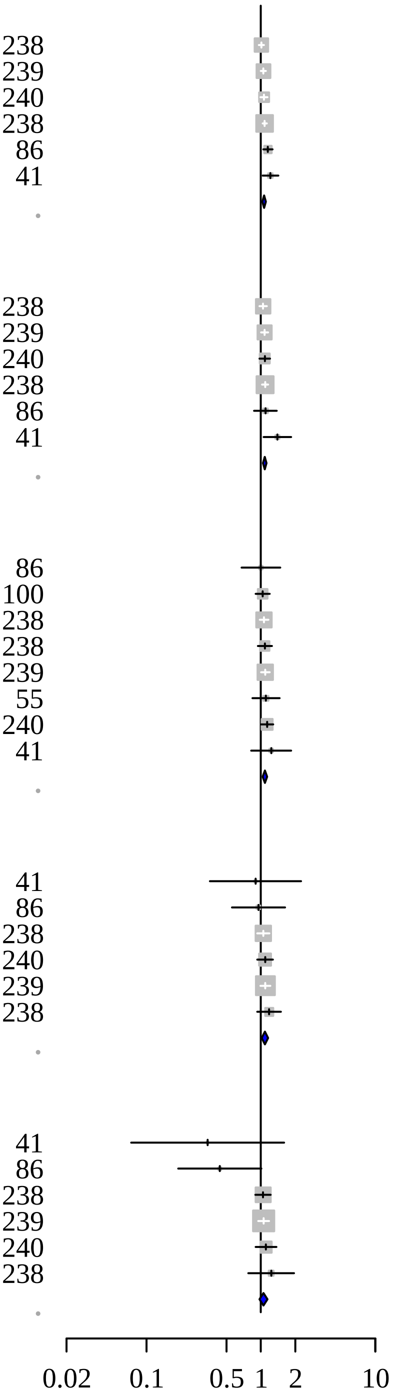

Appendix 7

Small study effects in three-year metastasis-free survival

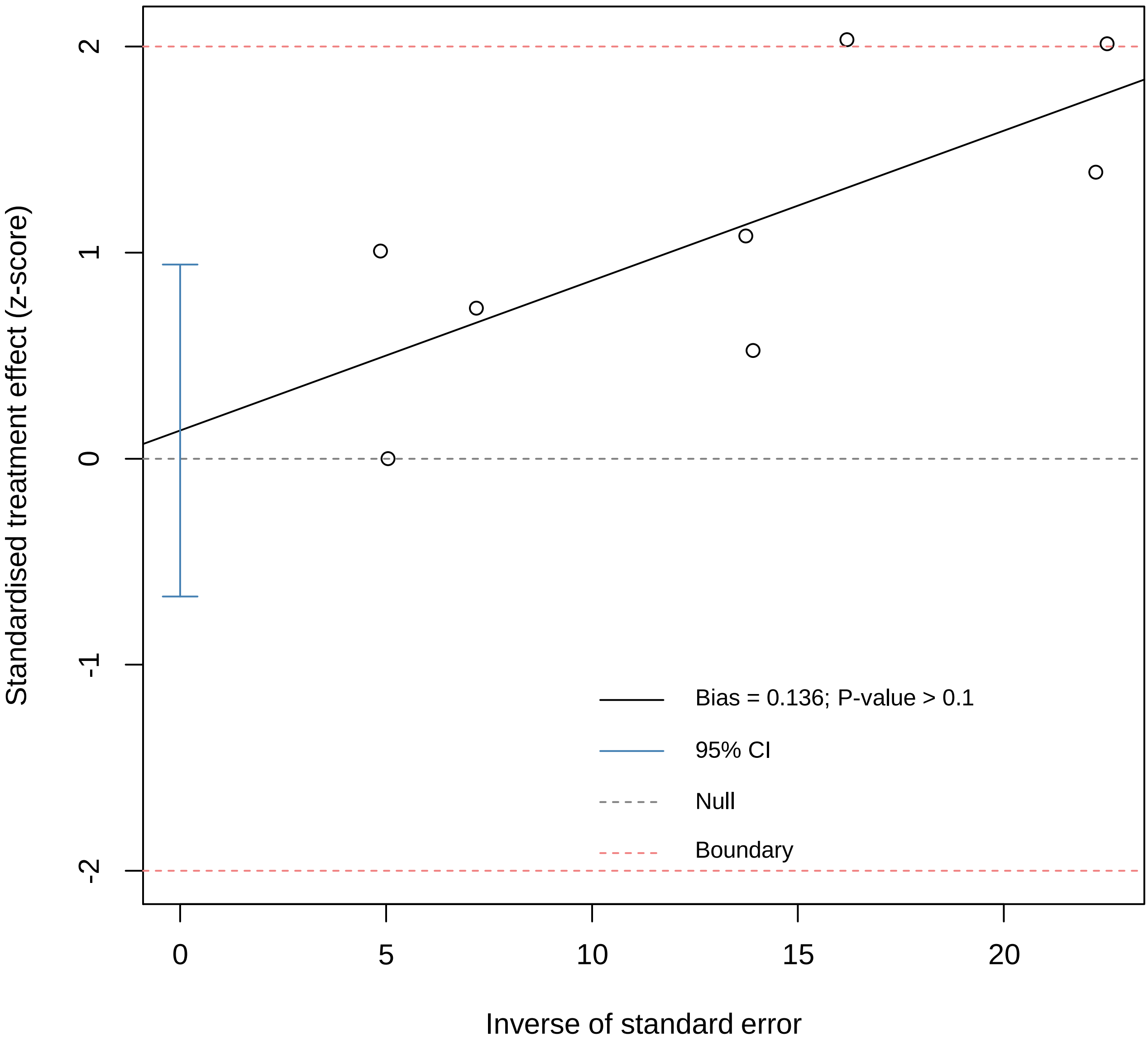

Appendix 8

Forest plot for local recurrence free survival

Study	CCRT Events	CCRT Total	IC-CCRT Events	IC-CCRT Total	RR	95%-CI
**FollowUp = 1**						
Yang et al. 2019	229	238	227	238	1.01	[0.97; 1.05]
Zhang et al. 2019	237	242	230	238	1.01	[0.98; 1.04]
Li et al. 2019	231	241	225	239	1.02	[0.98; 1.06]
Hong et al. 2018	218	239	200	240	1.09	[1.02; 1.17]
Frikha et al. 2017	39	40	31	41	1.29	[1.08; 1.55]
**Pooled effect**					1.02	[1.00; 1.04]
Heterogeneity: I^2 = 63%, p = 0.03						
**FollowUp = 2**						
Li et al. 2019	212	241	201	239	1.05	[0.97; 1.12]
Yang et al. 2019	217	238	207	238	1.05	[0.98; 1.12]
Hong et al. 2018	183	239	172	240	1.07	[0.96; 1.19]
Zhang et al. 2019	225	242	206	238	1.07	[1.01; 1.14]
Frikha et al. 2017	37	40	24	41	1.58	[1.20; 2.07]
**Pooled effect**					1.07	[1.03; 1.10]
Heterogeneity: I^2 = 54%, p = 0.07						
**FollowUp = 3**						
Huang et al. 2012	75	100	72	100	1.04	[0.88; 1.23]
Zhang et al. 2019	152	242	141	238	1.06	[0.92; 1.22]
Yang et al. 2019	204	238	192	238	1.06	[0.98; 1.15]
Li et al. 2019	203	241	188	239	1.07	[0.98; 1.17]
Gao et al. 2013	48	57	41	55	1.13	[0.93; 1.37]
Hong et al. 2018	166	239	146	240	1.14	[1.00; 1.30]
Frikha et al. 2017	24	40	20	41	1.23	[0.82; 1.84]
**Pooled effect**					1.08	[1.03; 1.13]
Heterogeneity: I^2 = 0%, p = 0.94						
**FollowUp = 4**						
Frikha et al. 2017	7	40	8	41	0.90	[0.36; 2.24]
Yang et al. 2019	170	238	160	238	1.06	[0.94; 1.20]
Li et al. 2019	193	241	171	239	1.12	[1.01; 1.24]
Zhang et al. 2019	97	242	81	238	1.18	[0.93; 1.49]
Hong et al. 2018	139	239	115	240	1.21	[1.02; 1.44]
**Pooled effect**					1.12	[1.05; 1.20]
Heterogeneity: I^2 = 0%, p = 0.74						
**FollowUp = 5**						
Frikha et al. 2017	1	40	6	41	0.17	[0.02; 1.36]
Yang et al. 2019	142	238	139	238	1.02	[0.88; 1.19]
Li et al. 2019	175	241	162	239	1.07	[0.95; 1.20]
Hong et al. 2018	102	239	91	240	1.13	[0.90; 1.40]
Zhang et al. 2019	36	242	27	238	1.31	[0.82; 2.09]
**Pooled effect**					1.07	[0.98; 1.16]
Heterogeneity: I^2 = 7%, p = 0.37						

Test for subgroup differences: χ^2 = 11.96, df = 4 (p = 0.02)

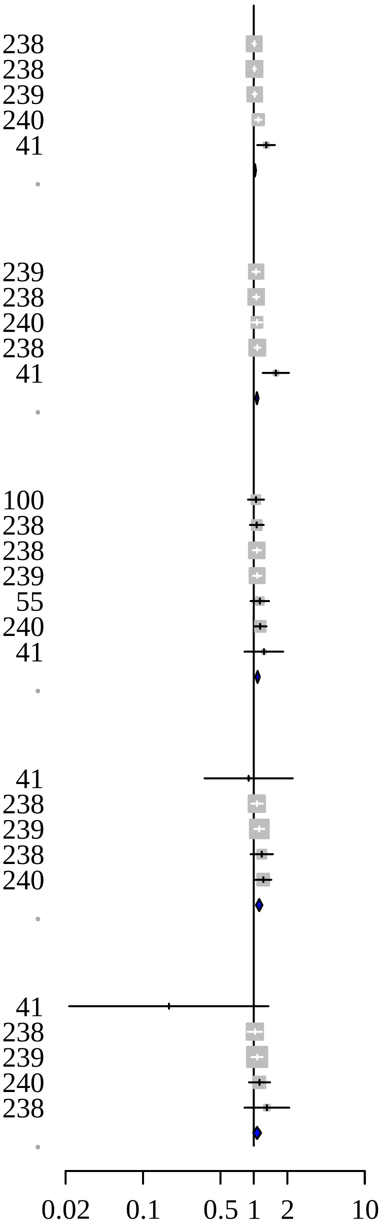

Appendix 9

GOSH analysis for two-year local recurrence free survival

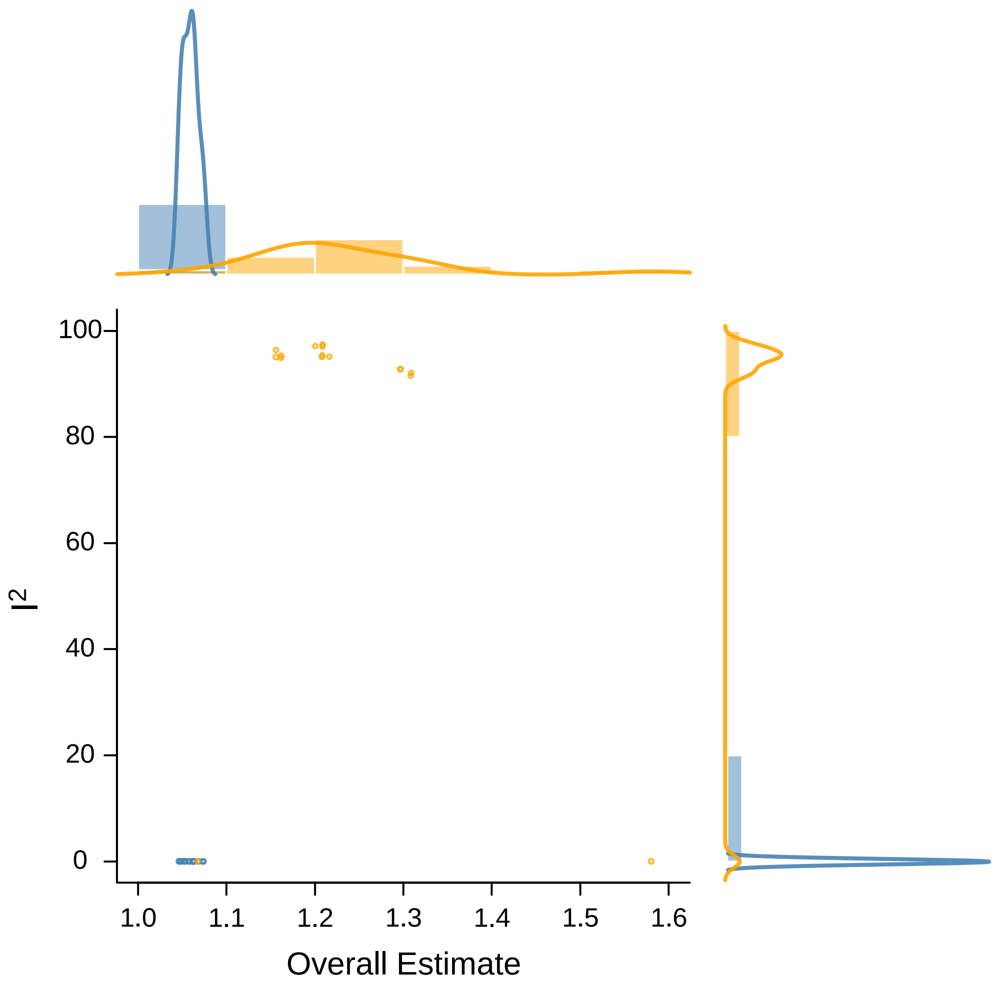

Blue: overall
Orange: trial by Frikha et al. was removed

Appendix 10

Small study effects in three-year local recurrence free survival

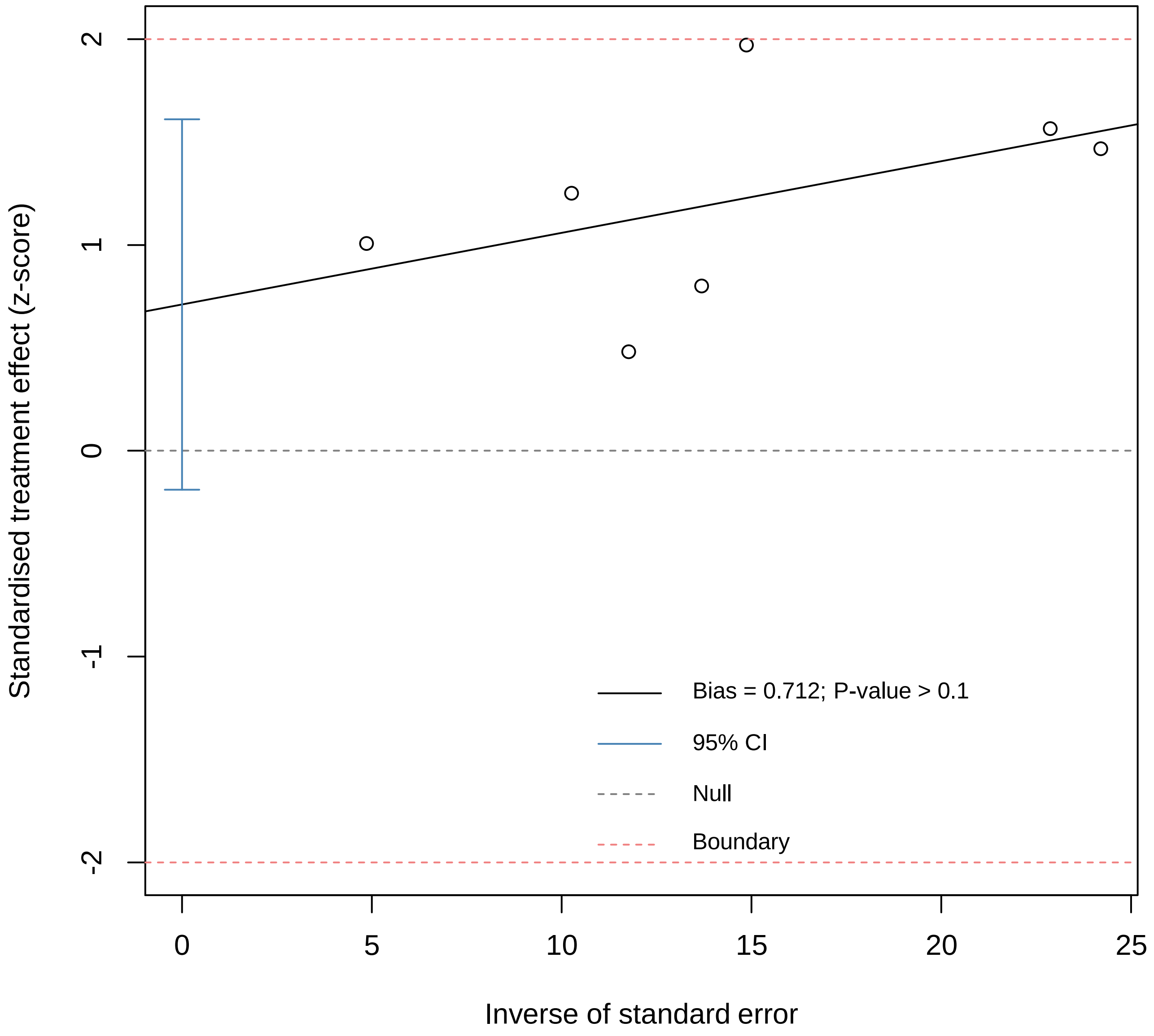

Supplement: Supplementary file 1 [file DataSheet_1.pdf]
